# Characterization of Different Sources of Human MSCs Expanded in Serum-Free Conditions with Quantification of Chondrogenic Induction in 3D

**DOI:** 10.1155/2019/2186728

**Published:** 2019-06-20

**Authors:** Hugo Fabre, Maxime Ducret, Olivier Degoul, Jonathan Rodriguez, Emeline Perrier-Groult, Elisabeth Aubert-Foucher, Marielle Pasdeloup, Céline Auxenfans, Colin McGuckin, Nico Forraz, Frédéric Mallein-Gerin

**Affiliations:** ^1^Laboratory of Tissue Biology and Therapeutic Engineering, CNRS UMR 5305, University Claude Bernard-Lyon 1 and University of Lyon, 7 Passage du Vercors, 69367 Lyon, France; ^2^CTI-BIOTECH, Cell Therapy Research Institute, 5 Avenue Lionel Terray, 69330 Meyzieu, France; ^3^Banque de Tissus et Cellules, Laboratoire des Substituts Cutanés, Hôpital Edouard Herriot, Hospices Civils de Lyon, Pavillon I, 5 Place d'Arsonval, 69347 Lyon, France

## Abstract

Mesenchymal stem cells (MSCs) represent alternative candidates to chondrocytes for cartilage engineering. However, it remains difficult to identify the ideal source of MSCs for cartilage repair since conditions supporting chondrogenic induction are diverse among published works. In this study, we characterized and evaluated the chondrogenic potential of MSCs from bone marrow (BM), Wharton's jelly (WJ), dental pulp (DP), and adipose tissue (AT) isolated and cultivated under serum-free conditions. BM-, WJ-, DP-, and AT-MSCs did not differ in terms of viability, clonogenicity, and proliferation. By an extensive polychromatic flow cytometry analysis, we found notable differences in markers of the osteochondrogenic lineage between the 4 MSC sources. We then evaluated their chondrogenic potential in a micromass culture model, and only BM-MSCs showed chondrogenic conversion. This chondrogenic differentiation was specifically ascertained by the production of procollagen IIB, the only type II collagen isoform synthesized by well-differentiated chondrocytes. As a pilot study toward cartilage engineering, we encapsulated BM-MSCs in hydrogel and developed an original method to evaluate their chondrogenic conversion by flow cytometry analysis, after release of the cells from the hydrogel. This allowed the simultaneous quantification of procollagen IIB and *α*10, a subunit of a type II collagen receptor crucial for proper cartilage development. This work represents the first comparison of detailed immunophenotypic analysis and chondrogenic differentiation potential of human BM-, WJ-, DP-, and AT-MSCs performed under the same serum-free conditions, from their isolation to their induction. Our study, achieved in conditions compliant with clinical applications, highlights that BM-MSCs are good candidates for cartilage engineering.

## 1. Introduction

Articular cartilage presents poor intrinsic repair capacity and traumatic and degenerative lesions of articular cartilage predisposed to osteoarthritis (OA), a worldwide leading source of disability. Current surgical options (microfracture, mosaicplasty) are not completely satisfactory since they often result in production of fibrocartilage. Besides, joint replacement is a short-term therapy for young individuals since knee prostheses have limited lifespan. Autologous chondrocyte implantation (ACI) was the first cell therapy applied to orthopedic surgery [1] and is the most widely used cell-based surgical procedure to treat focal damages of articular cartilage. However, ACI causes donor-site morbidity and this technique also involves a step of cell expansion that induces chondrocyte dedifferentiation [2], a process that is a major risk of producing fibrocartilage. The mesenchymal stem cells (MSCs) represent a promising alternative cellular reservoir for cartilage engineering since they can be easily isolated from different tissue sources and possess self-renewal and multipotency and have been described as being immunoprivileged cells with immunosuppressive properties. A recent study showing that MSCs isolated from bone marrow (BM) or adipose tissue (AT) maintain their immunosuppressive properties after chondrogenic differentiation [3] suggests that they might represent a cellular material of choice for off-the-shelf, allogenic strategies for cartilage engineering.

Different sources of MSCs have been studied in the literature with respect to their ability to convert into chondrocytes. Nevertheless, it remains difficult to identify the ideal tissue source of human MSCs for cartilage repair since the culture conditions and the cell model systems supporting chondrogenesis (pellet, micromass, hydrogel, natural, or synthetic scaffolds) used among research groups are diverse. In addition, most reported protocols refer to serum or animal products in the culture medium, preventing, therefore, clinical translation for cartilage therapies. The aim of this study was to investigate the chondrogenic potential of different categories of human MSCs using serum-/xeno-free, defined media from their isolation and expansion and during their chondrogenic induction. As MSCs derived from different tissue sources comprise several subpopulations that may possess specific functional characteristics [4], it is therefore absolutely necessary to deepen the molecular characterization of the different categories of MSCs that are proposed to be used in regenerative medicine. According to the minimum criteria defined originally by the International Society of Cellular Therapy (ISCT) to identify MSCs, cells should adhere to plastic, express specific cell-surface antigens CD73, CD90, and CD105, but not CD11b, CD14, CD19, CD34, CD45, CD79a, and HLA-DR, and be able to differentiate into osteoblasts, adipocytes, and chondrocytes *in vitro* [5]. However, these minimum criteria are not specific to MSCs and describe features shared by other connective tissue cells [6]. Considerable efforts have followed to extend MSC characterization using other surface markers but with great discrepancy or inconsistency mainly because of a lack of standardized conditions for the cell culture and immunophenotyping analysis. The first objective of this study was to undertake an extensive comparative polychromatic flow cytometric immunophenotyping of MSCs isolated from BM, AT, dental pulp (DP), and Wharton's jelly (WJ). In particular, we assessed expression of a panel of surface markers (here referred to as “advanced characterization” markers) that are described in the literature as being putative markers of skeletal precursor cells. These markers include CD15, CD49a, CD56, CD63, CD106, CD146, CD271, CD340, *α*10 integrin, and Stro-1. This is the first study providing detailed “identity cards” by analyzing in total 31 surface markers on MSCs isolated from BM, AT, DP, and WJ and expanded in conditions compliant with medicinal manufacturing. We also assessed the clonogenicity of these cells and their amplification kinetics in serum-free conditions.

The second objective of this study was to investigate the chondrogenic potential of BM-, AT-, DP-, and WJ-MSCs maintained in serum-free conditions. First, we used the micromass model, a 3D cell model largely used to mimic the mesenchymal cell condensation observed in the early phase of chondrogenesis during limb development [7, 8]. In this model, only BM-MSCs showed chondrogenic induction, as judged by expression of selected chondrogenic markers. Then, as a pilot study toward exploitation of the chondrogenic potential of MSCs for cartilage engineering, we combined BM-MSCs with a hydrogel scaffold to induce chondrogenesis and developed an original flow cytometry analysis using an antibody obtained and characterized in our laboratory [9] that selectively recognizes the IIB form of type II procollagen protein, a cartilage-specific form originally found to be expressed by well-differentiated human chondrocytes [10]. We demonstrate that BM-MSCs synthesize type IIB procollagen after chondrogenic induction in hydrogel, thus attesting to the quality of chondrocyte differentiation. Taken together, our data generated by using specific serum-/xeno-free culture conditions covering cell isolation, expansion, and chondrogenic induction of 4 different MSC types indicate that BM-MSCs appear as the most promising alternative cells to be clinically applied for cartilage regeneration.

## 2. Materials and Methods

### 2.1. Ethics Approval and Consent to Participate

All tissue specimens were collected upon written informed consent of the donors or their parents and complied with local ethical guidelines, national and European Union legislation regarding human sample collection, manipulation, and personal data protection. All experimental protocols were approved by the French Ministry of Higher Education and Research (Ethics Committee for research with human samples, CODECOH: DC-2014-2325).

### 2.2. Explant and Cell Culture during Amplification

For the isolation of cells by explant culture, explants were seeded in 6-well culture dishes and removed when cells reached confluence. For the isolation and amplification of MSCs, chondrocytes, and fibroblasts, all cell culture surfaces were precoated with a mixture of human collagens I and III (ABCell-Bio) at a concentration of 10 *μ*g/cm^2^ for isolation and of 50 *μ*g/cm^2^ for expansion. The cells were cultivated in serum-free SPE-IV defined medium (ABCell-Bio) containing 100 IU/mL penicillin and 100 *μ*g/mL streptomycin (P/S, Gibco, Life Technologies, Saint Aubin, France), at 37°C in a humidified atmosphere containing 5% CO_2_. The culture medium was changed 3 times a week. At confluence, cells were detached with a xeno-free recombinant protease (TrypLE Select 1X, Life Technologies) and seeded at 5 × 10^3^ cells/cm^2^ in flasks (Corning) for amplification. The cells were designated P0 during the isolation phase and P1 when reseeded after their first detachment. Subsequently, the number of passages designing cells (P2 to P10) corresponds to the number of detachments undergone by the cells.

### 2.3. AT-MSC Isolation

AT-MSCs were collected from 18 donors undergoing an abdominal liposuction for cosmetic purposes (age range: 28-67 years, median: 46 years; 40% male and 60% female). The AT was aspirated via a 3 mm blunt cannula, and AT-MSCs were isolated following two protocols. In the first protocol, AT was rapidly digested with collagenase A (0.120 U/mL, Roche) at 37°C for 30 min under constant shaking. Digestion was stopped by adding Dulbecco's modified Eagle's medium (DMEM) with GlutaMAX (Invitrogen) containing 10% clinical-grade fetal calf serum (FCS) (HyClone) supplemented with 1% P/S. Floating adipocytes were discarded, and cells from the stromal-vascular fraction were pelleted, rinsed with medium, centrifuged, and incubated in erythrocyte lysis buffer (Hybri-Max, Sigma-Aldrich) for 10 min at 37°C. After centrifugation and resuspension, cells were seeded at a density of 8 × 10^4^ cells/cm^2^. In the second protocol, AT aspirate was placed in a 50 mL tube containing 20 mL of phosphate-buffered saline (PBS) (Lonza) supplemented with 1% P/S. The mixture was shaken thoroughly to remove the remaining blood and potential microbial contaminants. The fat lobules (average size 1 cm^3^) were transferred to a new tube, and the procedure was repeated until the solution remained clear after agitation. The fat lobules were rinsed with PBS and then cut with a scalpel into 0.5 to 2 mm^3^ fragments to form explants. No differences in cell growth kinetics were observed between AT-MSCs isolated with the two protocols.

### 2.4. BM-MSC Isolation

BM was obtained from iliac crest aspirations of 10 donors (age range: 2-41 years, median: 13 years; 80% male and 20% female). The aspirate was washed once in PBS supplemented with 1% P/S and centrifuged at 1000 × g for 15 min. The cell pellet was resuspended in culture medium, and cells were seeded at 1.2 × 10^6^ cells/cm^2^. After 2 days, nonadherent cells were discarded by rinsing with PBS and adherent BM-MSCs were cultivated to confluence.

### 2.5. WJ-MSC Isolation

Umbilical cords were collected from 10 donors, before or after delivery of the placenta. A section of the umbilical cord between 10 and 25 cm long was cut and placed in a 50 mL tube containing 20 mL of PBS supplemented with 1% P/S. The mixture was shaken thoroughly to remove the remaining blood and potential microbial contaminants; then, the umbilical cord fragment was transferred to a new tube and the procedure was repeated until the solution remained clear after agitation. The cord fragment was transversally cut into 1 to 2 mm thick slices. Circular blades were then used to isolate standardized WJ pieces 2.5 mm in diameter to form explants.

### 2.6. DP-MSC Isolation

Teeth between Nolla developmental stages 5 and 8 were extracted from 10 donors, for orthodontic reasons (age range: 12-17 years, median: 15 years; 30% male and 70% female). Dental pulps were aseptically extirpated from the pulp chamber with fine tweezers. The apical part of the radicular pulp was removed with a scalpel to prevent contamination by dental papilla and periapical cells. The remaining pulp was then rinsed with PBS containing 1% P/S and cut with a scalpel into 0.5 to 2 mm^3^ fragments to form explants.

### 2.7. Fibroblast Isolation

Fibroblasts were obtained from foreskin biopsies collected from 10 donors undergoing surgical circumcisions (age range: 2-8 years, median: 5 years). The biopsy was placed in a 50 mL tube containing 20 mL of PBS supplemented with 1% P/S. In order to separate dermis and epidermis, biopsy was incubated with 25 mL of 0.25% trypsin (Sigma-Aldrich) at 37°C for 1 to 1.5 h. After enzymatic digestion, dermis and epidermis could be separated using 2 sterile tweezers. The dermis was further processed as for WJ-MSCs: a biopsy punch was used to isolate standardized tissue pieces that were 2.5 mm in diameter and 1 to 2 mm thick to form explants.

### 2.8. Chondrocyte Isolation

Chondrocytes were extracted from macroscopically healthy zones of articular cartilage obtained from 5 donors undergoing total knee replacement (age range: 50-77 years, median: 68.5 years; 40% male and 60% female) or from nasal cartilage of 7 donors undergoing rhinoseptoplasty (age range: 50-72 years, median: 51 years; 30% male and 70% female). Small slices of cartilage were sequentially digested with 0.2% trypsin (Sigma-Aldrich) for 30 min at 37°C, followed by 0.15% bacterial collagenase A (Roche Applied Science) supplemented with 1% P/S overnight. The cells were then seeded at a density of 0.8 × 10^4^ cells/cm^2^.

### 2.9. Cell Viability, Proliferation Kinetics, and Clonogenic Potential

The cell viability was estimated by flow cytometry analysis. The nucleic acid dye 7-amino-actinomycin D (7-AAD; BD Biosciences, Le Pont-de-Claix, France) was used for the exclusion of nonviable cells.

The cells were counted using a Cellometer (Nexcelom) at each passage. The population doubling (PD) level was calculated according to following the equation: PD = [log10(NH) − log10(NS)]/log10(2), where NH is the number of harvested cells and NS is the number of seeded cells. The cumulative population doubling (CPD) level was obtained by adding the PD levels of the previous passages: CPD = ∑(PD). The doubling time (DT) was calculated following the equation DT = CT/PD, where CT is the cell culture time.

The colony-forming unit fibroblast (CFU-F) assays were performed by seeding P1 cells in two replicas at a density of 100 cells/well in 6-well plates. After 14 days, cells were fixed with ice-cold methanol and stained with 0.3% (*w*/*v*) crystal violet (Sigma-Aldrich). Visible colonies were quantified only when they were isolated and greater than or equal to 50 cells.

### 2.10. Polychromatic Flow Cytometry Analysis

#### 2.10.1. Sample Processing

About 1 million P1 cells (MSCs, fibroblasts, and chondrocytes) were harvested for analysis. To prevent unspecific binding of antibodies to the cell surface, cells were preincubated with Human TruStain FcX Fc Receptor Blocking Solution (BioLegend) for 10 min at room temperature, before staining with specific antibodies. The cells were stained with 31 fluorochrome-conjugated antibodies and with 7-AAD to exclude nonviable cells. The list of antibody clones used in this study is presented in Supplementary Figure 1, and the different combinations of antibodies coupled with their fluorochromes are described in Supplementary Figure 2.

#### 2.10.2. Data Acquisition

Samples were acquired on a BD FACSCanto II Flow cytometer (BD Biosciences) equipped with 3 lasers (violet 405 nm, blue 488 nm, and red 633 nm) as uncompensated events and recorded as FCS 3.0 files. Analysis and compensation were performed using FlowJo software. As gating strategy, a primary Boolean gate was placed on 7-AAD-negative cells. This population was then visualized on the height versus width signal of the side scatter (SSC-H/SSC-W) and on the height versus width signal of the forward scatter (FSC-H/FSC-W) dot plot to exclude doublets and clumps. A final gate was placed on the area versus width signal of the forward scatter (FSC-A/FSC-W) dot plot to discriminate the cell population from the remaining debris. This whole gating process enabled analysis of a viable single cell population. The percentage of cells positively stained corresponded to the percentage of cells present within a gate established so that less than 1% of the measured positive events represented nonspecific binding by the fluorochrome-conjugated isotype-matched control. In addition, fluorescence minus one (FMO) controls were used in combination with isotype controls (Supplementary Figure 3). Autofluorescence (when observed) was excluded by leaving a blank detector in the violet laser line, and autofluorescent cells were either compensated as regular fluorochrome or gated out of the analysis.

### 2.11. Analysis of Procollagen IIB and *α*10 Integrin by Flow Cytometry

Rabbit polyclonal antibodies to human procollagen IIB (pNIIB52) [9] were fluorescently labeled using Zenon Alexa Fluor 647 Rabbit IgG Labeling Kit (Invitrogen), according to the manufacturer's instructions. The resulting antibodies are referred as AF647-IIB procollagen antibodies. Each cell-agarose construct was dissolved with 500 *μ*L of 100 U/mL agarase (Sigma-Aldrich) in PBS at 37° for 1 h. The cell suspension was washed twice with PBS before resuspension in staining buffer containing bovine serum albumin (BSA) (BD Biosciences). To prevent unspecific binding of antibodies to the cell surface, 1 × 10^6^ cells were incubated with Human TruStain FcX Fc receptor blocking solution (BioLegend) for 10 min at room temperature, before staining with Fixable Viability Dye eFluor 450 (FVD-eFluor 450) (Bioscience) for 1 h at 4°C, according to the manufacturer's instructions. The cells were then incubated with 1 *μ*g of anti-*α*10 integrin primary antibody (a kind gift from Dr. Evy Lundgren-Akerlund, Xintela AB, Sweden) for 1 to 2 h at 4°C, washed twice with stain buffer, and incubated for 1 to 2 h with goat anti-mouse IgG2a secondary antibody conjugated to Alexa Fluor 488 (AF488; Invitrogen). The cells were then washed twice with stain buffer, fixed, and permeabilized using BD Cytofix/Cytoperm solution (BD Biosciences) following the manufacturer's instructions and stained with AF647-IIB procollagen antibodies for 1 h at 4°C. Acquisitions were performed in a BD FACSCanto II flow cytometer (BD Biosciences) and analyzed using FlowJo software.

### 2.12. Osteogenic and Adipogenic Differentiation

All reagents were purchased from Sigma-Aldrich unless otherwise specified. Osteogenesis and adipogenesis were induced in monolayer cultures of MSCs, fibroblasts, and chondrocytes, after 2 passage amplification in serum-free SPE-IV defined medium. The cells were initially plated at a density of 1 × 10^4^ cells/cm^2^ in 6-well plates, and differentiation was performed for 21 days, when cells reached confluence.

Osteogenic medium consisted of high-glucose DMEM (Gibco) supplemented with 10% fetal bovine serum (FBS), 10^−7^ M dexamethasone, 50 *μ*g/mL L-ascorbate-2-phosphate, 10 mM *β*-glycerophosphate, and 1% P/S antibiotics. On day 21, cultures were fixed in 60% isopropanol and stained with 1% *w*/*v* alizarin red S solution (ARS) to detect matrix mineralization. For ARS staining quantification, 10% acetic acid solution was added to the wells for 30 min, cells were scraped with a cell scraper, and each cell suspension was transferred to a 1.5 mL microcentrifuge tube, heated at 85°C for 10 min, and centrifuged. The supernatant was transferred to a new tube, and acid was neutralized by addition of 0.4 volume of 10% ammonium hydroxide. Aliquots were deposited in triplicate in a 96-well plate, and absorbance was read at 405 nm with a microplate reader.

Adipogenic medium consisted of high-glucose DMEM (Gibco) supplemented with 10% FBS, 1 *μ*M dexamethasone, 100 *μ*M indomethacin, 0.5 mM 3-isobutyl-1-methyl-xanthine, 1% P/S antibiotics, and 200 mUI/mL insulin (Umulin, Lilly laboratories). On day 21, cells were fixed with 10% formalin (Merck), rinsed with water, and then incubated with 60% isopropanol. Isopropanol was removed, and intracellular accumulation of lipid vacuoles was visualized by staining with 0.2% *w*/*v* oil red O for 10 min followed by rinsing with water. For quantification, oil red O stain was extracted by adding 100% isopropanol; then, aliquots were deposited in triplicates in a 24-well plate and absorbance was read at 492 nm with a microplate reader.

### 2.13. Chondrogenic Differentiation

All reagents were purchased from Sigma-Aldrich unless otherwise specified. P1 MSCs and fibroblasts amplified in serum-free SPE-IV defined medium were used for chondrogenic induction.

For pellet induction, 3.5 × 10^5^ cells were seeded in V-bottomed 96-well plates and centrifuged for 10 min at 250 g. The pellets were cultivated for 28 days in high-glucose DMEM supplemented with 1% P/S, 1 mM sodium pyruvate (Gibco), 50 *μ*g/mL L-ascorbate-2-phosphate, 0.1 *μ*M dexamethasone, 1% ITS (insulin-transferrin-selenium, Gibco) in the absence (control medium) or presence of 50 ng/mL recombinant human BMP-2 (dibotermin-alpha, drug form of BMP-2, InductOs kit, Wyeth) and 10 ng/mL recombinant human TGF-*β*3 (R&D Systems). This latter medium was referred as BT*β*3 medium. P1 chondrocytes were used as positive control of chondrogenic differentiation. The chondrocytes were amplified and induced in pellet either by using the same culture conditions as for MSCs and fibroblasts or by supplementing serum-free SPE-IV medium with 5 ng/mL FGF-2 (R&D Systems) and 5 *μ*g/mL insulin (UMULINE RAPIDE, Lilly) during amplification and with 200 ng/mL BMP-2, 5 *μ*g/mL insulin, and 100 nM tri-iodothyronine (T3) during induction. These amplification and differentiation media were designated FI medium and BIT medium, respectively. The culture media were changed every day for the first three days and then every 2-4 days.

For chondrogenic induction in hydrogel, BM-MSCs were embedded in 2% agarose (SeaPlaque, Cambrex Bioscience). Briefly, 100 *μ*L agarose disks containing 9 × 10^5^ cells were shaped by molding agarose suspension in precut 1 mL pipette tips. After gelling, the constructs were moved to 24-well culture plates. The disks were then cultivated for 21 days in BT*β*3 medium supplemented or not with 5% serum-free SPE-IV medium. The culture media were changed every day for the first three days and then every 2-4 days. The cell viability was evaluated with the Cellstain double staining kit.

### 2.14. Histological and Immunohistochemical Analyses

The pellets and agarose gels were rinsed in PBS and fixed for 24 h with 4% neutral-buffered formalin. After dehydration, samples were embedded in paraffin and sectioned at 5 *μ*m. To detect sulfated proteoglycans, sections were stained with Safranin O in 0.1 M sodium acetate (pH 7.4) for 10 min. For immunohistochemistry, sections were digested with 0.5% hyaluronidase diluted in PBS containing 3% bovine serum albumin (PBS-BSA) for 1 h to unmask the antigenic sites. Incubation with primary antibodies was carried out in PBS-BSA overnight at 4°C. Polyclonal rabbit antibodies to human type II collagen (Novotec, Ref. 20211) were used at 1 : 500. Polyclonal rabbit antibodies to human type I collagen (Novotec, Ref. 20111) were used at 1 : 1000. Polyclonal rabbit antibodies specific to human procollagen IIB (anti-pNIIB52) and raised in our laboratory [9] were used at 1 : 250. After washing with PBS and PBS with 0.2% Tween-20, endogenous peroxidase activity was inhibited by treatment with 0.5% aqueous H_2_O_2_ in PBS-BSA. Then, anti-rabbit horseradish peroxidase-conjugated secondary antibodies (Dako, Ref. K4002), used undiluted, were applied for 45 min. Finally, sections were revealed with diaminobenzidine, counterstained with hematoxylin and eosin, and observed with a DM 4000B microscope (Leica) directly coupled to a color camera (Digital Camera DXM1200, Nikon). Image acquisition was achieved with MetaView software (Universal Imaging).

### 2.15. Gene Expression Analysis

Total RNA was isolated from MSCs, fibroblasts, and chondrocytes that were cultivated in monolayer (P1) or in pellets for 21 days. Three pellets for each culture condition and for each donor were pooled for RNA extraction. Total RNA was extracted by using the RNeasy Mini kit (QIAGEN). Reverse Transcription (RT) was performed using from 80 to 500 ng total RNA with PrimeScript RT Reagent Kit (Takara) according to the manufacturer's instructions. Real-time PCR amplification was performed in a 20 *μ*L reaction mix containing 10 *μ*L FastStart Universal SYBR Green Master (Roche), 4 *μ*L cDNA (1 : 3 dilution), 300 nM primers, and 4 *μ*L water. Amplification was performed in a Rotor-Gene Q cycler (QIAGEN). Cycling conditions consisted of a denaturation step at 95°C for 2 min, 40 cycles of 95°C for 15 s, and annealing and extension at 60°C for 30 s. The primer sequences are listed in Supplementary Figure 4. *COL2A1* and *ACAN* encode characteristic proteins of native hyaline cartilage, and *COL1A1*, *COL10A1*, and *MMP-13* encode extracellular matrix (ECM) molecules or enzymes of other cartilage types. *ALPL* and *RUNX2* encode bone markers. *LEP* and *PPARG* encode markers of adipose tissue. Housekeeping genes were *GAPDH* for pellet analysis and *GUSB* for osteogenesis and adipogenesis analyses. Each assay was performed in duplicate, and mRNA relative quantification was done using the 2^-ΔΔCt^ method.

### 2.16. Statistical Analysis

Data were generated with cells derived from at least three donors, and *n* represents the number of donors in the figure legends. Statistical analysis was carried out using GraphPad Prism software (version 5.00; GraphPad Software, San Diego, CA, USA). Data are presented as mean ± standard deviation (SD) or box plots. Normally distributed samples with *n* ≥ 5 (amplification kinetics, flow cytometry data) were compared using the one-way analysis of variance followed by post hoc Tukey's multiple comparison test. Normally distributed samples with *n* = 3 (analysis of stain quantification and gene expression in trilineage differentiation studies) were compared to control with a paired *t*-test. Probability values (*p*) inferior to 0.05 (^∗^, ^#^), 0.01 (^∗∗^, ^##^), or 0.001 (^∗∗∗^, ^###^) were considered to be statistically significant and marked in the figures accordingly.

## 3. Results

### 3.1. Characteristics of MSCs in Serum-Free Culture

**Figure 1:**
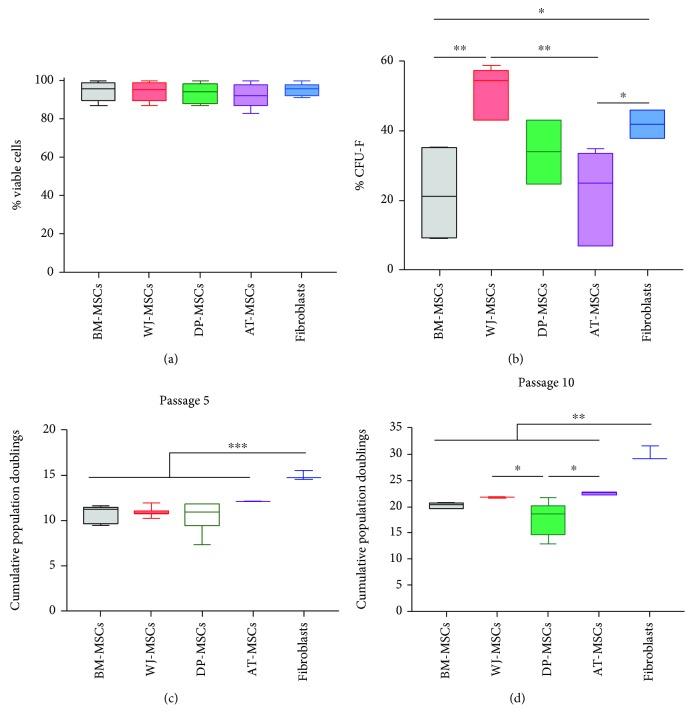
Characterization of MSCs, in comparison with fibroblasts. (a) Determination of cell viability estimated at passage 0 (*n* = 8 for BM- and WJ-MSCs, *n* = 5 for DP- and AT-MSCs, and *n* = 3 for fibroblasts). (b) Quantification of colony-forming unit fibroblasts (CFU-Fs) evaluated at passage 1 (*n* = 5 for BM-, WJ-, DP-, and AT-MSCs and *n* = 3 for fibroblasts). The cell proliferation was estimated by determining the cumulative population doubling levels (c) at passage 5 and (d) at passage 10, as indicated (*n* = 5 for BM-MSCs, *n* = 6 for WJ-MSCs, *n* = 8 for DP-MSCs, *n* = 4 for AT-MSCs, and *n* = 3 for fibroblasts). Data are presented as box plots with median as a bar. ^∗^Statistically significant differences (^∗^ *p* < 0.05, ^∗∗^ *p* < 0.01, and ^∗∗∗^ *p* < 0.001).

We first determined the viability, clonogenicity, and proliferation capacities of BM-, WJ-, DP-, and AT-MSCs in comparison with fibroblasts, the major connective tissue cell type. The mean viability of the cells at the end of the isolation phase (passage 0, P0) was 94.51 ± 4.75% for BM-MSCs, 94.19 ± 4.65% for WJ-MSCs, 93.3 ± 5.33% for DP-MSCs, 92.34 ± 6.25% for AT-MSCs, and 95.14 ± 3.36% for fibroblasts (Figure 1(a)). Morphological examination revealed that all these cell types were spindle shaped and fibroblastic in appearance (data not shown). The colony-forming unit fibroblasts (CFU-Fs) are considered to represent the frequency of MSCs in a cell population when colonies are generated at clonal density [11]. Because the majority of cells isolated from BM are hematopoietic nonadherent cells whereas the majority of cells isolated from WJ, DP, and AT represent nonhematopoietic, adherent cells, CFU-F assays were performed with cells that were passaged once (P1 cells). This timing of analysis allowed BM-MSCs to adhere and allowed us subsequently to use the same clonal density for all the categories of MSCs. The percentage of CFU-Fs was the highest for WJ-MSCs (51 ± 7.4%) and it was 34 ± 9.2% for DP-MSCs, similar for BM-MSCs and AT-MSCs (22 ± 13% and 21 ± 13.4%, respectively), while it was 42 ± 4% for fibroblasts (Figure 1(b)).

The cells were passaged up to 10 times, and population doublings (PD), cumulative population doublings (CPD), and doubling times (DT) were calculated. The DT remained relatively stable for all cell sources between P1 and P5 and increased afterwards except for the fibroblasts that retained their proliferative capacities (Supplementary Figure 5). At P5, fibroblasts showed the highest CPD (15 ± 0.5) while BM-MSCs, WJ-MSCs, DP-MSCs, and AT-MSCs showed close CPD (10.74 ± 0.97, 11.02 ± 0.5, 10.49 ± 1.51, and 12.10 ± 0.09, respectively) (Figures 1(c) and 1(d)). The gap between fibroblasts and MSCs increased at P10 with fibroblasts showing again the highest CPD (30 ± 1.45) and BM-MSCs, WJ-MSCs, DP-MSCs, and AT-MSCs showing CPD values fairly similar (20.23 ± 0.53, 21.83 ± 0.17, 17.70 ± 3.19, and 22.53 ± 0.28, respectively).

### 3.2. Immunophenotypic Characterization of MSCs, Fibroblasts, and Chondrocytes after Expansion in Serum-Free Conditions

**Figure 2:**
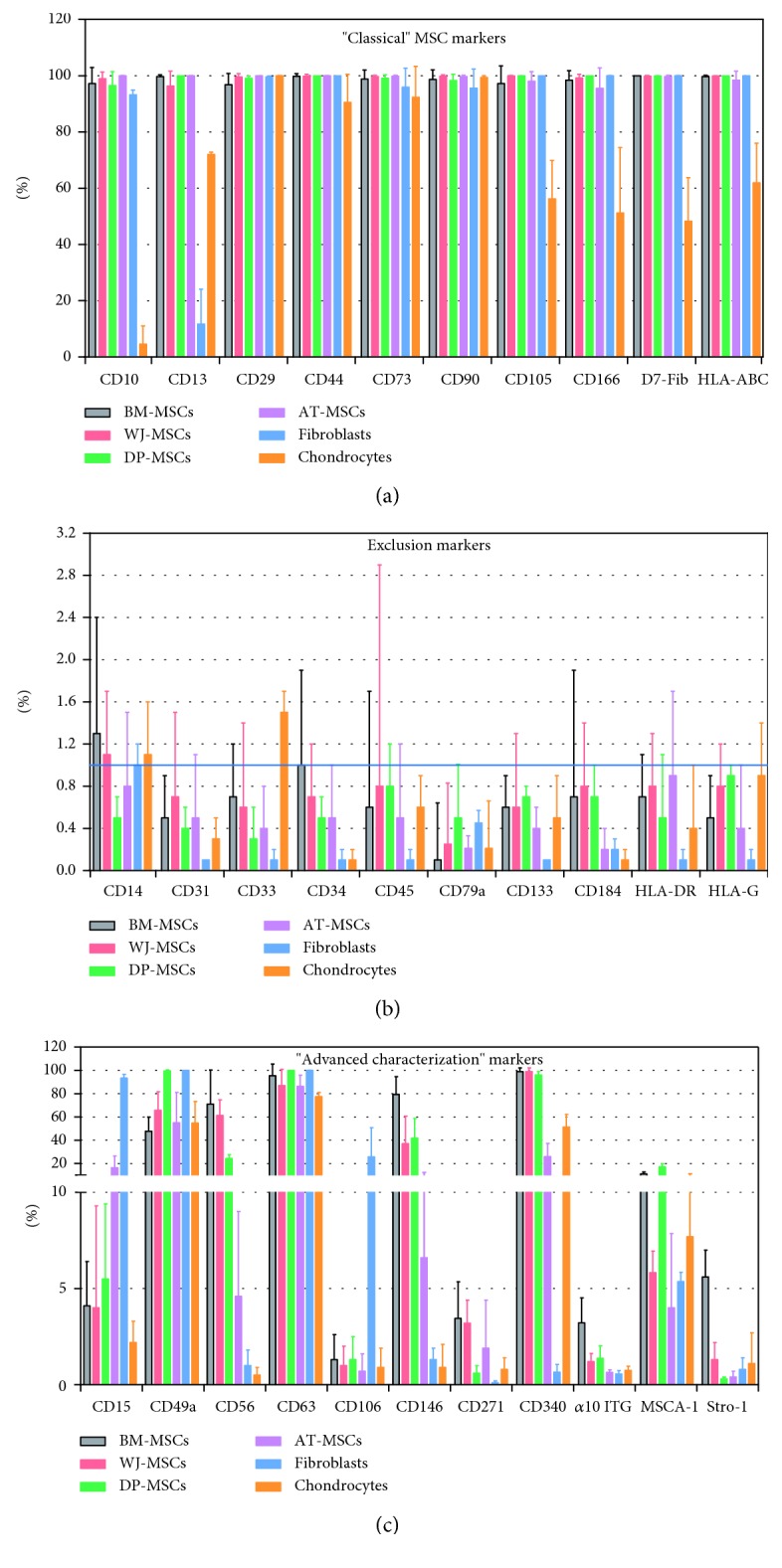
Comparative immunophenotypic analysis of MSCs, fibroblasts, and articular chondrocytes cultivated under serum-free conditions. A total of 31 cell markers were analyzed at passage 1, by using multicolor flow cytometry. (a) “Classical” MSC markers, (b) exclusion markers, and (c) “advanced characterization” markers (*n* = 8 for BM-MSCs, *n* = 5 for DP-, WJ-, and AT-MSCs, and *n* = 3 for fibroblasts and chondrocytes). The blue line indicates the 1% threshold. Error bars: mean ± SD.

A comparative immunophenotypic analysis of MSCs, fibroblasts, and chondrocytes cultivated at passage 1 (P1) was performed by flow cytometry with a panel of 31 cell surface markers. The results presented in Figure 2(b) and Supplementary Figure 6 showed that for all cell sources, the mean percentage of cells expressing markers characteristic of hematopoietic cells (CD34, CD45, and CD133), endothelial cells (CD31), blood-circulating progenitor cells (CD184), monocytes and macrophages (CD14), myeloid progenitors (CD33), B cells (CD79a), antigen-presenting cells (HLA-DR), and immune tolerance-associated cells (HLA-G) was in overall below the 1% threshold, or just above. These markers were referred to as exclusion markers (Figure 2(b) and Supplementary Figure 6). On the opposite, all MSC sources and fibroblasts were positive for markers originally referred to as MSC markers by the ISCT in 2006 (CD73, CD90, and CD105) and for other surface MSC markers proposed more recently (CD10, CD13, CD29, CD44, CD166, D7-Fib, and HLA-ABC). We classified this first series of positive MSC markers as “classical” markers (Figure 2(a) and Supplementary Figure 6). It is worth noting that all MSC sources but only a subset of fibroblasts (11.7 ± 12.4%) shared the expression of CD13. The chondrocytes were positive for several MSC markers (CD29, CD44, CD73, and CD90), but only subsets of chondrocytes were positive for CD10 (4.6 ± 6.4%), CD13 (72 ± 0.8%), CD105 (56.2 ± 13.7%), CD166 (51.2 ± 23.3%), D7-Fib (48.3 ± 15.4%), and HLA-ABC (61.9 ± 14.1%). We further explored the expression of “advanced characterization” markers, selected from the literature as being putative markers of the osteochondrogenic lineage (Figure 2(c) and Supplementary Figure 6). Only two of them, CD49a and CD63, were expressed by the majority of cells from all tissue sources. The other markers varied in their expression depending on the cell source. Stro-1, *α*10 integrin, and CD271 were expressed to a low frequency in all cell sources while CD15, CD56, CD106, CD146, CD340, and MSCA-1 showed expression that appeared more restricted to specific cell sources. Of note, CD340 was absent from the fibroblast population whereas CD15 was the only marker expressed preferentially by fibroblasts (93.3 ± 3.3%) in comparison with BM-MSCs, WJ-MSCs, DP-MSCs, AT-MSCs, and chondrocytes that were stained weakly positive for this marker (4.1 ± 2.3%, 4 ± 5.3%, 5.5 ± 3.9%, 16.4 ± 10.1%, and 2.2 ± 1.1%, respectively).

### 3.3. Osteogenic and Adipogenic Differentiation Capacities of MSCs

We first investigated the potential of the MSCs to differentiate into osteoblasts and adipocytes *in vitro* in comparison with that of fibroblasts and chondrocytes (Supplementary Figure 7(a)). After exposure to osteogenic medium, signs of extracellular matrix mineralization were detected in all cell sources, as judged by alizarin red S staining, while control cells cultivated in standard growth medium remained negative. Quantification of alizarin red staining revealed that the level of mineralization was the lowest for fibroblasts and the highest for chondrocytes. To better characterize the differentiation stage of the cells, mRNA expression of *RUNX2* (coding for the Runt-related transcription factor 2, a master gene of osteogenesis) and that of *ALPL* (coding for alkaline phosphatase, a bone matrix protein) were examined. *RUNX2* was stimulated under osteogenic condition in all MSC sources, although to variable levels, whereas no significant differences in the levels of *RUNX2* expression were noted in fibroblasts and chondrocytes between control and osteogenic conditions. *ALPL* was stimulated in osteogenic condition in all cell sources except WJ- and DP-MSCs.

Regarding adipogenic differentiation (Supplementary Figure 7(b)), all cell sources except fibroblasts showed lipid droplets in their cytoplasm under adipogenic conditions and quantification of oil red O staining confirmed that fibroblasts were the only cell type to present no sign of adipogenesis. We also looked for mRNA expression of *PPARG*, a key regulator of adipocyte differentiation coding for the peroxisome proliferator-activated receptor gamma and of *LEP*, coding for the adipokine leptin. All MSC categories showed significant but variable inductions of *PPARG*. This induction remained modest in chondrocytes and was absent in fibroblasts. All cell sources except fibroblasts and WJ-MSCs showed significant induction of *LEP* in adipogenic conditions.

### 3.4. Chondrogenic Differentiation Capacity of MSCs in Pellet Cultures under Serum-Free Conditions

**Figure 3:**
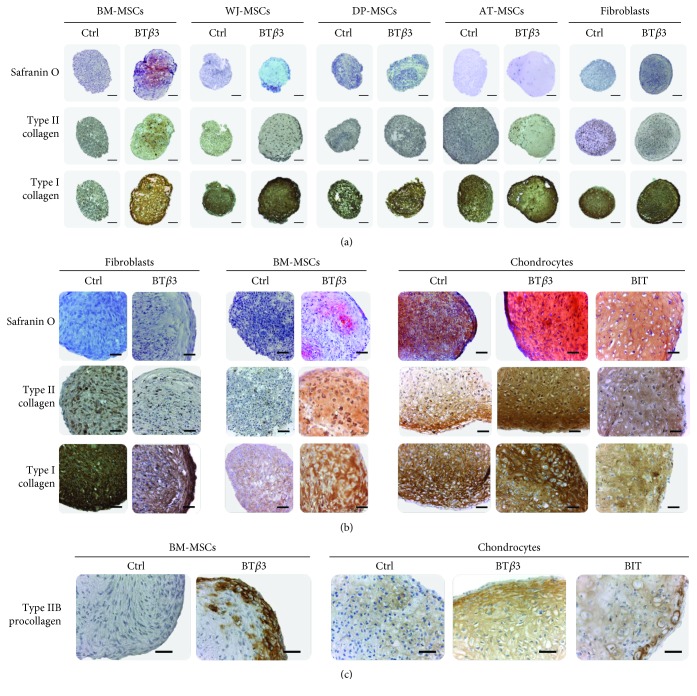
Histological and immunohistochemical evaluation of MSC pellets cultivated for 28 days under serum-free conditions, in control medium (Ctrl) or in chondrogenic medium (BT*β*3 or BIT), as indicated. (a) Pellets from BM-MSCs, WJ-MSCs, DP-MSCs, AT-MSCs, and fibroblasts. Adjacent paraffin sections were stained with Safranin O or analyzed immunohistochemically for type I collagen and total type II collagen. The scale bars represent 200 *μ*m. (b) Comparison of BM-MSC pellets with pellets from fibroblasts and nasal chondrocytes, representing negative and positive control of chondrogenesis, respectively. Adjacent paraffin sections were stained with Safranin O or analyzed immunohistochemically for type I collagen and total type II collagen, as indicated. The scale bars represent 50 *μ*m. (c) Comparison of BM-MSC pellets with chondrocyte pellets. Adjacent sections were analyzed immunohistochemically for type IIB procollagen. The scale bars represent 50 *μ*m.

We next compared the chondrogenic differentiation potential of the different sources of MSCs after amplification and chondrogenic induction under serum free-conditions. Pelleted MSCs were cultivated in the absence (control medium) or in the presence of BMP-2 and TGF-*β*3 (chondrogenic BT*β*3 medium) for 28 days. Safranin O staining, revealing proteoglycans, and type II collagen immunostaining demonstrated promotion of the chondrogenic phenotype only in BM-MSCs whereas type I collagen production was induced in all MSC categories (Figure 3(a)). Noticeably, type I collagen immunostaining appears to be weaker in BM-MSCs, when compared with the other MSC sources and fibroblasts (a cell source classically known for robust type I collagen expression) treated with chondrogenic medium as negative control of chondrogenesis (Figure 3(a)).

In order to better evaluate the degree of chondrogenic differentiation reached by the BM-MSCs, chondrocytes were also cultivated in pellets as positive controls of chondrogenesis. The chondrocyte pellets were induced with BT*β*3 medium or with a culture medium (referred to as BIT medium) containing a cocktail of soluble factors (BMP-2, insulin, and thyroxin T3) originally shown to be efficient for inducing human auricular or articular chondrocytes to produce a cartilage matrix in collagen biomaterials [12–14]. The chondrocyte pellets showed strong Safranin O staining and type II collagen immunostaining after culture with BT*β*3 or BIT medium while these stainings appeared weaker and sparser in control medium (Figure 3(b)). On parallel sections, the chondrocytes showed homogenous and comparable levels of type I collagen immunostaining in control and chondrogenic media. The BM-MSC pellets were stained positive for Safranin O and type II collagen only upon induction with BT*β*3 medium, with sparse staining for Safranin O and homogenous staining for type II collagen (Figure 3(b)). The BM-MSCs were also positive for type I collagen after induction with BT*β*3 medium. The intensity of this staining was equivalent with that seen with chondrocytes but much weaker than that seen with fibroblasts (Figure 3(b)).

Type II collagen can be synthesized as two isoforms, a nonchondrogenic IIA form and a cartilage-specific IIB form. A shift from IIA to IIB forms has been reported to occur at the mRNA level during the chondrogenic differentiation of MSCs originated from different sources [15, 16]. Thus, type IIB procollagen represents a unique marker of well-differentiated chondrocytes. We recently characterized the first antibody (referred as anti-pNIIB52) able to selectively detect the IIB form of human type II procollagen in Western blot or IHC analysis [9]. Here, we used this tool to refine the chondrogenic status of the BM-MSCs by immunostaining. The chondrocyte pellets were stained clearly positive for procollagen IIB after induction with the two chondrogenic media, with stronger staining at the periphery of the pellets (Figure 3(c)). The BM-MSC pellets were also stained positive for procollagen IIB, and this staining appeared restricted mainly to the peripheral zone of the pellets. After culture in control medium, chondrocytes showed weak staining and BM-MSCs showed no staining for procollagen IIB (Figure 3(c)).

**Figure 4:**
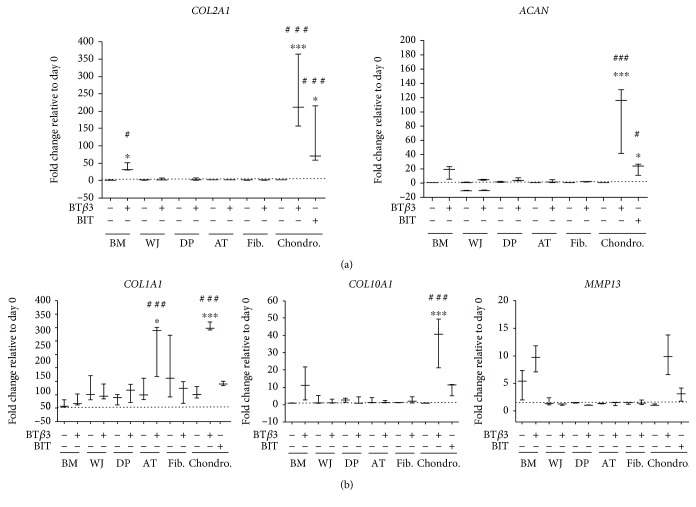
Effect of culture conditions on the mRNA steady-state levels of gene markers of chondrocyte differentiation. Pellets of MSCs, fibroblasts, and nasal chondrocytes were cultivated for 21 days under serum-free conditions in control medium (as indicated by -) or in chondrogenic medium containing BT*β*3 or BIT cocktail (as indicated by +). The values obtained in pellets are expressed relative to P1 cells amplified in monolayer under serum-free conditions and used as a reference before differentiation (day 0, reference value = 1). Data are presented as box plots with median as a bar (*n* = 3). ^∗^Statistically significant differences between BT*β*3- and BIT-treated cells and control cells (^∗^ *p* < 0.05, ^∗∗^ *p* < 0.01, and ^∗∗∗^ *p* < 0.001). ^#^Statistically significant differences between cells cultivated in pellets and day 0 cells (^#^ *p* < 0.05, ^##^ *p* < 0.01, and ^###^ *p* < 0.001).

In order to complement these histological observations at the gene level, we next specified the differentiation status of the MSCs, fibroblasts, and chondrocytes, by monitoring mRNA levels at the end of chondrogenic induction in pellet cultures. These mRNA levels were compared with those of control medium-treated cells or cells at the end of the amplification phase in the monolayer (day 0). We first analyzed expression of two chondrocyte marker genes, *COL2A1* coding for type II collagen and *ACAN* coding for the core protein of aggrecan, the major cartilage proteoglycan. A significant upregulation of *COL2A1* was observed in BT*β*3- and BIT-treated chondrocytes (over 244- and 114-fold, respectively) and in BT*β*3-treated BM-MSCs (37-fold), relatively to control or day 0 cells (Figure 4(a)). Similarly, *ACAN* was found significantly upregulated in BT*β*3- and BIT-treated chondrocytes (96- and 21-fold, respectively) and weakly upregulated in BT*β*3-treated BM-MSCs (16-fold), in comparison with control or day 0 cells (Figure 4(a)).

We also analyzed *COL1A1*, coding for the *α*1 chain of type I collagen. BT*β*3 treatment significantly upregulated expression of *COL1A1* in AT-MSCs (236-fold relatively to day 0 and 3.7-fold relatively to control cells) and in chondrocytes (303-fold relatively to day 0 and 5.3-fold relatively to control cells) (Figure 4(b)).

Since a main challenge of using MSCs for cartilage engineering is to prevent MSC-derived chondrocytes from undergoing hypertrophic maturation, we next looked for expression of two genes characteristic of hypertrophic chondrocytes, *COL10A1* coding for type X collagen and *MMP-13* coding for matrix metalloproteinase-13. No significant upregulation of *COL10A1* or *MMP-13* was found in BT*β*3-treated MSCs and fibroblasts, in comparison with day 0 or control cells (Figure 4(b)). The only significant upregulation was found for *COL10A1* (37-fold) in BT*β*3-treated chondrocytes, when compared with control or day 0 cells (Figure 4(b)).

### 3.5. Chondrogenic Induction of BM-MSCs in Hydrogel under Serum-Free Conditions

**Figure 5:**
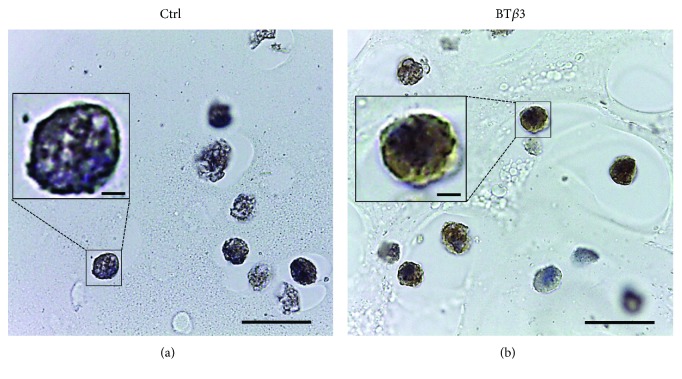
Immunohistochemical staining for total type II collagen in BM-MSC/agarose constructs. After expansion in monolayer under serum-free conditions, P1 BM-MSCs were seeded in agarose. The constructs were then cultivated under serum-free conditions for 21 days in control medium (Ctrl) or in chondrogenic medium (BT*β*3). Paraffin sections were revealed with diaminobenzidine and counterstained with hematoxylin and eosin. Cells that are negatively or positively stained for type II collagen appear blue or brown, respectively (scale bars represent 10 *μ*m). Insets show cells at higher magnification (scale bars represent 1 *μ*m).

As a next step towards cartilage engineering based on hydrogel and MSCs, we evaluated the chondrogenic potential of BM-MSCs embedded and cultivated in agarose, under serum-free conditions. First, we found that the serum-free conditions (DMEM high glucose + 1%ITS + BT*β*3) used for chondrogenic differentiation of MSCs in pellets were not able to support the viability of the BM-MSCs in agarose but supplementation of this chondrogenic medium with 5% of SPE-IV serum-free medium maintained good viability, as shown after 10 days of culture (Supplementary Figures 8(a) and 8(b)). We also used chondrocytes as reference control of chondrogenesis in this hydrogel. Of note, chondrocyte viability was not affected in agarose in the presence of the serum-free BT*β*3 or BIT culture medium (data not shown). We then examined chondrogenic differentiation of BM-MSCs after 21 days of culture in agarose, by immunohistochemical staining for type II collagen. Most BM-MSCs were positive for type II collagen in chondrogenic medium while they remained negative in control medium (Figure 5). This first observation seemed to indicate that BM-MSCs could engage in chondrogenesis in agarose.

### 3.6. Development of an Original Quality Control of Chondrogenic Conversion in Hydrogel by Flow Cytometry

Since the main theme of this study was to investigate if MSCs can represent reliable alternative candidates to chondrocytes in cartilage transplantation strategies, it was important to determine if BM-MSCs embedded in hydrogel are capable of fully differentiating into chondrocytes, after chondrogenic induction in serum-free conditions. With this aim, we used anti-pNIIB52 and flow cytometry to estimate the frequency of type IIB procollagen-expressing cells in the population of BM-MSCs embedded in agarose. First, we developed experimental conditions to validate the use of anti-pNIIB52 in flow cytometry. A titration assay was performed by incubating chondrocytes with different concentrations of anti-pNIIB52 labeled with Alexa Fluor 647 dye, in order to detect IIB expression with optimal signal to noise ratio (Supplementary Figure 9(a)). We then used fluorescently labeled anti-pNIIB52 to analyze IIB procollagen expression in dedifferentiated or redifferentiated chondrocytes. After amplification in monolayer, chondrocytes were negative to IIB procollagen whereas redifferentiation triggered by 21 days of BT*β*3 treatment resulted in 37.6 ± 4.2% or 94.6 ± 2.1% of positive cells, for chondrocytes redifferentiated in monolayer or in agarose, respectively (Supplementary Figure 9(b)). The higher percentage of procollagen IIB-expressing chondrocytes observed in agarose was consistent with the well-known 3D effect of agarose on cytoskeletal organization and re-establishment of the differentiation state of chondrocytes, independently of the role of growth factors [17]. Furthermore, to establish staining specificity of anti-pNIIB52 in flow cytometry, we used the immunizing peptide that was originally shown to block anti-pNIIB52 in Western blotting and immunohistochemistry analyses [9]. Indeed, well-differentiated chondrocytes remained negative to IIB procollagen when fluorescently labeled anti-pNIIB52 was first preincubated with the immunizing peptide (Supplementary Figure 9(c)). In addition, we confirmed by using imaging flow cytometry that anti-pNIIB52 recognizes type IIB procollagen in intracellular vesicles (data not shown). This series of pilot experiments conducted with chondrocytes demonstrated that anti-pNIIB52 was a suitable and specific tool to recognize IIB procollagen by flow cytometry.

**Figure 6:**
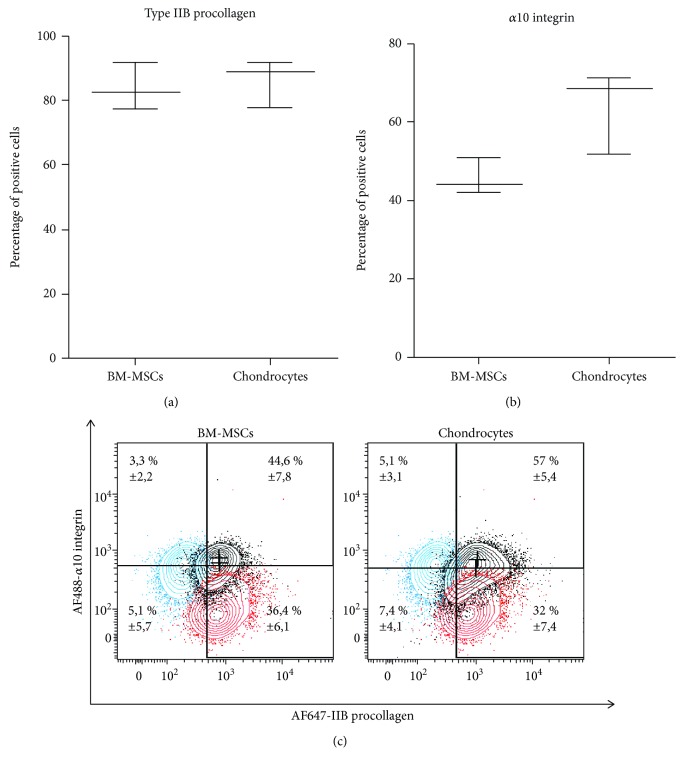
Flow cytometry analysis of *α*10 integrin and IIB procollagen expression. After expansion in monolayer under serum-free conditions, P1 BM-MSCs and nasal chondrocytes were seeded in agarose hydrogel. The cell-agarose constructs were then cultivated under serum-free conditions for 21 days in the presence of BT*β*3 (for BM-MSCs) or BIT (for chondrocytes). The cells were released from the constructs by agarase digestion and analyzed by flow cytometry. Percentage of cells expressing (a) type IIB procollagen or (b) *α*10 integrin. Data are presented as box plots with median as a bar (*n* = 3). (c) Single-stained populations (*α*10^−^/IIB^+^ and *α*10^+^/IIB^−^) and double-stained populations (*α*10^+^/IIB^+^) are present in relatively equivalent proportions in BM-MSCs and chondrocytes, as reported in the quadrants (percentage of positive cells is represented as mean ± SD, *n* = 3). Red contour: IIB procollagen; blue contour: *α*10 integrin.

Our next step was to analyze BM-MSCs by flow cytometry, after 21 days of culture in agarose in the presence or absence of BT*β*3. For both culture conditions, a mortality rate of about 50% was observed when BM-MSCs were released from hydrogel and stained with FVD-eFluor 450 fixable viability dye (data not shown). Of note, signs of cell death were not observed when chondrocytes were released from agarose (data not shown), suggesting that BM-MSCs were particularly sensitive to agarase treatment and/or loss of their pericellular environment. The dead cells were gated out of the analysis based on FVD-eFluor 450 staining, and we compared expression of type IIB procollagen with that of the *α*10 integrin subunit, one of the “advanced characterization” markers analyzed by flow cytometry in this study. Integrins are transmembrane proteins consisting of *α* and *β* subunits, and *α*10*β*1 integrin was originally identified as a collagen-binding receptor on chondrocytes [18]. A more recent study has shown that *α*10 is expressed at low levels by BM-MSCs in monolayer culture and that *α*10 expression increases during *in vitro* chondrogenesis in pellet culture [19]. Very interestingly, the same study has reported that FGF-2-induced upregulation of *α*10 in BM-MSCs in monolayer culture enhances subsequently chondrogenesis and synthesis of type II collagen and aggrecan in pellet culture [19]. Thus, *α*10 can be considered as a quality marker of chondrocytes and predictive marker of MSCs with chondrogenic potential. Our flow cytometry analysis revealed that BT*β*3-treated BM-MSCs and BIT-treated chondrocytes expressed relatively comparable levels of type IIB procollagen (83.3 ± 9.2% for BM-MSCs and 86.3 ± 7.6% for chondrocytes) and *α*10 (45.3 ± 5.1% for BM-MSCs and 61.2 ± 11.4% for chondrocytes), after 21 days of culture in agarose (Figure 6(a)). Regarding coexpression of IIB procollagen and *α*10, BM-MSCs and chondrocytes displayed similar profiles with a major double-stained *α*10^+^/IIB^+^ population (44.6 ± 7.8% for BM-MSCs and 57 ± 5.4% for chondrocytes (Figure 6(b)). Further, single-stained populations were also clearly visible among BM-MSCs and chondrocytes, with an *α*10^−^/IIB^+^ population (36.4 ± 6.1% for BM-MSCs and 32 ± 7.4% for chondrocytes) and a more minor *α*10^+^/IIB^−^ population (3.3 ± 2.2% for BM-MSCs and 5.1 ± 3.1% for chondrocytes) (Figure 6(b)). These results together revealed that type IIB procollagen and *α*10 expressions were not strictly correlated.

## 4. Discussion

MSCs have already entered the clinical arena, and they are increasingly considered for cell-based tissue engineering for cartilage repair. They may provide an alternative to chondrocytes, but their properties, including the ability of chondrogenesis, are reported to depend on their source [4, 20, 21]. In this study, we compared the characteristics of BM-, WJ-, DP-, and AT-MSCs by a detailed flow cytometry analysis of cell surface markers and their potential to differentiate into chondrocytes by using 3D cultures. In view of their clinical potential, we strove to perform this analysis using serum-/xeno-free cell culture medium.

A reliable MSC-based approach for tissue engineering first implies efficient cell isolation and expansion. Our cell isolation procedures used with BM, WJ, DP, and AT allowed high rates of cell viability and our cell culture conditions ensured constant cell doubling times, from P1 to P5. The overall decrease in cell proliferation observed after the fifth passage is consistent with previous studies showing that MSCs have a limited lifespan and enter senescence after a certain number of cell divisions [22]. Interestingly, for each category of MSCs, we harvested a minimum of 10^6^ cells at P1 from one donor, a number consistent with that required for hydrogel-based cartilage tissue engineering. For instance, in a pilot study aiming at reconstructing permanent cartilage applicable for clinical use, Liu et al. [13] created columnar implants 10 mm in diameter and 1 mm in thickness by adding 10^6^ auricular chondrocytes to atelocollagen gel combined to poly(L-lactic acid) scaffold. In addition, we calculated that a minimum of 300 × 10^6^ cells (for BM-MSCs) and up to 800 × 10^6^ cells (for AT-MSCs) could be obtained after 5 passages, starting with 10^6^ cells. This short-term expansion should allow genomic stability. In fact, by using the same cell culture conditions as here, we recently showed that DP-MSCs retain a normal karyotype after 4 passages [23]. Thus, the cell isolation and amplification procedures used in this study represent an efficient medicinal manufacturing approach to produce MSCs. Specifically, we showed that this approach can be beneficial for cartilage regeneration since expansion over one passage already generates a cell number sufficient to create a cartilage implant.

As a first step in the extensive characterization of MSCs derived from the different tissue sources, we analyzed their immunophenotype by flow cytometry, in comparison with fibroblasts and chondrocytes. We chose to analyze the cells at early passage (P1) since we were concerned that prolonged amplification could differentially modify cell surface marker expression in the different MSC sources. As we were also concerned that inappropriate cell-detaching methods could alter cell immunophenotype, we avoided the use of enzymes for isolating MSCs from tissues, as exemplified by our explant cultures. Moreover, to harvest cells in culture, we used the animal-origin free, recombinant dissociating enzyme TrypLE that has been shown to be more respectful of the cell surface antigens than the animal trypsin commonly used [24]. First, our results showed that all MSC sources, fibroblasts, and chondrocytes failed to express MSC exclusion markers originally defined by the ISCT (CD14, CD34, CD45, CD79a, and HLA-DR). We also showed that these three cell categories failed to express other surface antigens more recently excluded from MSCs, CD31, and CD33 [25]; CD133 [26]; CD184 [27]; and HLA-G [28]. On the other hand, all MSC sources expressed the ISCT-recommended markers (CD73, CD90, and CD105) and other markers now widely recognized as MSC markers in the literature: CD10 [29], CD13 and CD29 [30], CD44 [31], CD166 [32], D7-Fib [33], and HLA-ABC [30]. Furthermore, our comparative analysis revealed that a large proportion of fibroblasts and chondrocytes express several of these “classical” MSC markers, which is in line with previous studies showing that fibroblasts and chondrocytes share surface markers utilized for MSC characterization [34–36].

A major goal of this study was to compare the capacities of MSCs to engage chondrogenesis after amplification in the same conditions. Thus, we extended our immunophenotypic analysis to additional, more “advanced characterization” markers presented in the literature as being promising to identify cellular precursors of the osteochondrogenic lineage. CD15 is a carbohydrate molecule that has been identified at the surface of MSCs present in the periodontal ligament [37]. CD49a corresponds to the *α*1 integrin subunit and has been found associated with MSCs from synovium [38]. CD56 was identified as a neural cell adhesion molecule [39] and MSCA-1 as a tissue nonspecific alkaline phosphatase [40]. Interestingly, Battula et al. [41] have shown that the subpopulation of BM-MSCs coexpressing CD56 and MSCA-1 presents strong chondrogenic differentiation capability whereas the MSCA-1^+^/CD56^−^ subpopulation can differentiate into adipocytes only. CD63 is a glycoprotein member of the tetraspan transmembrane family originally found to be expressed by BM-MSCs and bone cells [42]. More recent studies have shown that CD63 is also expressed by chondrocytes [35, 43]. CD106 (vascular cell adhesion molecule 1 (VCAM-1)) and CD271 (nerve growth factor receptor (NGFR)) are expressed by MSCs derived from synovial membranes (SM), and Arufe et al. [44] have shown that the capacity to form chondrogenic spheroids was superior with CD271- and CD73-enriched SM-MSCs, in comparison with CD106-enriched SM-MSCs. Along the same lines, Mifune et al. [45] have shown that CD271^+^ BM-MSCs have greater ability than nonsorted BM-MSCs to form chondrogenic pellets *in vitro* and to restore cartilage after transplantation of the pellets in chondral defects created in rats. CD146 is a transmembrane glycoprotein belonging to the immunoglobulin family, and Sacchetti et al. [46] found that CD146^+^ cells, but not CD146^−^ cells, derived from BM-MSCs were osteogenic *in vivo* and could reestablish the hematopoietic microenvironment in a xenotransplantation model. Furthermore, a recent study combining flow cytometry, microarray, and functional analysis has revealed that CD146 and CD166 are expressed by prechondrogenic mesenchymal cells in developing human limb buds and that transition of mesenchymal cells to differentiated chondrocytes is associated with their loss [47]. CD340 (human epidermal growth factor 2 (HER2)) was among additional markers for BM-MSCs described about a decade ago [48]. More recently, it has been shown that the level of CD340 expression depends on the composition of the culture medium used to expand BM-MSCs and that addition of FGF-2 increases its expression and chondrogenic differentiation potential of these cells [49]. STRO-1 is a surface marker initially identified in BM stromal cells. It distinguishes a clonogenic cell fraction that can differentiate into osteoblasts, chondrocytes, adipocytes, or smooth muscle cells [50]. Interestingly, a recent study has reported robust expression of hyaline cartilage-specific markers by STRO-1^+^ cells immunoselected from BM and combined with porous polystyrene membranes. However, hypertrophic differentiation was not prevented indicating that strategies of *in vitro* chondrogenesis avoiding this phenotyping drift are still needed [51]. The integrin subunit *α*10 closes this list of “advanced characterization” markers. Globally, our analysis revealed wider variations in expression of these markers between the different cell sources, in comparison with the more “classical” MSC markers. These data indicate that BM-, WJ-, DP-, and AT-MSCs, cultivated rigorously in the same conditions, are not totally equivalent in their profiles of cell surface antigens. Whether these differences in surface phenotype correspond to different abilities of MSCs to engage chondrogenesis requires larger investigations. It is widely recognized that the use of cocktails rather than single antibodies will provide the most effective means of isolating specific precursors among MSC populations. Our extended dataset offers valuable information to explore further the benefits of cell selection for tissue engineering applications. Indeed, the results of our multiparametric flow cytometry analysis allow determination of coexpression of markers that could be exploited to isolate MSC subpopulations and evaluate their differentiation potentials.

The ability of BM-, WJ-, DP-, and AT-MSCs to differentiate toward osteoblasts and adipocytes in the presence of serum was demonstrated in this study, with variable efficiency depending on the cell source. These four types of MSCs have also been shown to maintain their chondrogenic ability after amplification in the presence of serum, as reported by us or other groups [32, 52–63]. Here, when serum-free conditions were specifically chosen from the isolation to the chondrogenic induction of MSCs, we found that only BM-MSCs succeeded in maintaining their chondrogenic potential. This chondrogenic capacity was unequivocally ascertained by production of the cartilage-specific IIB isoform of type II procollagen. The preferential distribution of type IIB procollagen observed in IHC images at the periphery of the pellets most likely results from a gradient of cell differentiation and/or collagen maturation occurring from the peripheral zone to the central core of the pellets. In addition, our real-time PCR studies showed induction of *COL2A1* and *ACAN* expression in BM-MSCs but did not reveal upregulation of *COL10A1* and *MMP-13* expression, indicating that hypertrophic maturation did not occur in the pellets. In concordance with this, we did not detect signs of cellular hypertrophy in the pellets. Further, the intensities of immunostaining and the levels of gene expression observed for type I collagen in BM-MSCs and in chondrocyte pellets appeared equivalent. These results together suggest that our culture conditions promote mainly a chondrocyte-characteristic phenotype in BM-MSCs. The reason why only BM-MSCs in our hands respond to chondrogenic induction is unclear although it could conceivably be related to their intrinsic properties. A recent study comparing MSCs derived from BM, AT, skin, and umbilical cord revealed that only BM-MSCs form spontaneously a hematopoietic niche *in vivo* through a vascularized cartilage intermediate [64]. In the same study, a restricted set of genes involved in skeletal development, including *ITGA10*, was found significantly upregulated and hypomethylated in BM-MSCs in culture, suggesting that this MSC source is specifically primed for skeletal development [64]. In this context, it is worth mentioning that the highest level of *α*10 in our flow cytometry analysis was found in BM-MSCs, in comparison with the other cell sources even with chondrocytes (Figure 2). Still, the proportion of *α*10^+^ BM-MSCs remained of modest value but this can be related to the low concentration of FGF-2 (0.33 ng/mL) present in SPE-IV medium [23]. In support of this, Varas et al. [19] showed that amplification of BM-MSCs for 2 weeks in the presence of 10% FBS generated 70% of *α*10^+^ cells provided that they were treated with 10 ng/mL FGF-2 whereas only 10% of cells were *α*10^+^ in the absence of FGF-2 treatment. Other studies have also shown that the chondrogenic potential of MSCs depends on the culture conditions. For instance, equivalent chondrogenic differentiation capacities have been found for BM- and AT-MSCs in serum-supplemented medium [65] whereas BM-MSCs showed higher chondrogenic differentiation potential than AT-MSCs when they were cultivated with human platelet lysate as a substitute to FBS [66]. Thus, our study reinforces the general view that it is crucial to identify the most suitable MSC source according to the culture process envisaged for a given clinical application.

For successful cartilage engineering, the use of a scaffold may be advantageous to maintain the transplanted cells in the defect. As a next step toward clinical application, we combined BM-MSCs with a hydrogel to support chondrogenesis under serum-free culture conditions. Hydrogels are good candidates for cartilage repair since they are 3D polymer networks rich in water and up to 80% of articular cartilage wet weight consists of water [67]. Here, we used agarose, a natural polymer that shows strong capacity to support the chondrocyte phenotype and cartilage matrix production [68, 69]. Interestingly, agarose has already been used to construct cartilage patches in clinical trials [70]. Our microscopic observations showed that most BM-MSCs were stained intracellularly for type II collagen after 3 weeks of chondrogenic induction in agarose, indicating good cell anabolic activity despite the serum-free conditions. However, there was no sign of extracellular type II collagen, suggesting that production of a structured ECM was not yet achieved within the timeframe of this experiment. In fact, a known limitation in using porous scaffolds is that they fail to accumulate most of the ECM proteins until the pericellular matrix is sufficiently developed [71]. Our flow cytometry analysis of BM-MSCs released from agarose further confirms that the cells acquired a phenotype typical of well-differentiated chondrocytes, as judged by the levels of expression of type IIB procollagen and the *α*10 integrin subunit, found to be in the same range as for true chondrocytes. In this investigation, we report the efficacy of anti-pNIIB52 for flow immunocytometry analysis. We believe that this antibody can be of great interest to evaluate the quality of chondrogenic conversion of MSCs since it detects the intracellular precursor form of a cartilage-specific matrix protein. Of note, after chondrogenic induction, about a third of BM-MSCs were *α*10^−^ but synthetized procollagen IIB (as observed with chondrocytes). This result probably reflects the fact that the presence of the *α*10 subunit at the surface of cells is not necessary for the production of this chondrocyte marker. In any case, evaluation of coexpression of *α*10 and procollagen IIB strengthens the quality assurance of the chondrocyte phenotype.

Here, in a relatively short *in vitro* study, we demonstrated the suitability of the agarose hydrogel as a 3D matrix to support chondrogenic differentiation of BM-MSCs in defined serum-free culture conditions. Longer studies are now required to determine if a sufficient cartilage matrix can be produced by BM-MSCS in these conditions and if agarose can be envisaged as a cell carrier for MSC-based cartilage repair. More generally, we think that the cell/agarose model, combined to flow immunocytometry analysis with known or candidate chondrogenic markers, may serve as a useful platform to optimize chondrogenic cultivation conditions or to identify the most suitable cell sources for cartilage engineering applications. Furthermore, mechanical conditioning has been reported to stimulate chondrogenic induction of MSCs [72, 73]. Therefore, the cell/agarose model could be used to monitor chondrogenesis under mechanical stimulation, as we have shown for chondrocytes [68].

## 5. Conclusions

This investigation represents the first head-to-head comparison of detailed immunophenotypic analysis and chondrogenic differentiation potential of BM-, WJ-, DP-, and AT-MSCs performed under serum-free conditions. We have reported an unprecedented coverage of 31 cell surface markers which contribute to refine molecular description of these MSCs. Our flow cytometry analysis showed that most “classical” MSC markers are expressed by BM-MSCs, WJ-MSCs, DP-MSCs, AT-MSCs, fibroblasts, and chondrocytes and this is in line with the general view that connective tissue cells and MSCs share similar biological characteristics. However, we found notable differences between MSC sources, based on the expression levels of several “advanced characterization” markers described as potential markers of the osteochondrogenic lineage. Whether these differences reflect different capabilities to engage chondrogenesis under serum-free conditions needs further studies, and the profiles of expression that we have identified can be exploited for sorting experiments. Importantly, we were able to generate, after short-term expansion, MSCs in sufficient number to substitute chondrocytes in a hydrogel plug of volume clinically relevant for cartilage repair. It will be interesting to determine the number of passages that MSCs can tolerate without adversely affecting their chondrogenic potential. Thus, our study performed in conditions compliant with medicinal manufacturing highlights that MSCs are a reliable cell source for cartilage tissue engineering. BM-MSCs appear as best candidates, but we do not exclude that MSCs from other tissue sources could present equivalent chondrogenic potential, in the presence of defined serum-/xeno-free cell culture conditions different than those presented here. We should also say that other factors such as donor sex and age are known to influence cell expansion and differentiation capacities of MSCs [74, 75] but our small-size cohorts of donors in our study preclude this type of analysis. Therefore, it will be interesting to supplement our work by studies with large tissue samples in order to assess the impact of cell sex and age on the chondrogenic potential of MSCs that are isolated, amplified, and induced in serum-free conditions.

## Data Availability

All data generated or analyzed during this study are included in this published article and the Supplementary Materials.

## Abbreviations

ACI: Autologous chondrocyte implantation
ARS: Alizarin red S solution
AT: Adipose tissue
BM: Bone marrow
BMP-2: Bone morphogenetic protein-2
CFU-F: Colony-forming unit fibroblast
CPD: Cumulative population doubling
DP: Dental pulp
DT: Doubling time
FBS: Fetal bovine serum
FMO: Fluorescence minus one
ISCT: International society of cellular therapy
ITS: Insulin-transferrin-selenium
MSCs: Mesenchymal stem cells
OA: Osteoarthritis
WJ: Wharton's jelly
PCR: Polymerase chain reaction
PD: Population doubling
PS: Penicillin streptomycin
RT: Reverse transcription
SM: Synovial membranes
T3: Tri-iodothyronine
TGF: Transforming growth factor.

## Supplementary Materials

Supplementary Materials Supplementary Figure 1: list of antibodies used for the immunophenotypic analysis. Supplementary Figure 2: presentation of the different combinations of antibodies coupled to fluorochromes that were used for the immunophenotypic analysis. Supplementary Figure 3: representative flow cytometric histograms of “classical,” exclusion, and “advanced characterization” markers expressed on BM-MSCs cultivated at passage 1, under serum-free conditions. Supplementary Figure 4: oligonucleotide primers used for the real-time polymerase chain reaction analysis [76]. Supplementary Figure 5: growth characteristics of MSCs and fibroblasts during long-term expansion under serum-free conditions. Supplementary Figure 6: immunophenotype of MSCs, fibroblasts, and chondrocytes cultivated under serum-free conditions. The expression of 31 markers was analyzed by flow cytometry. Supplementary Figure 7: evaluation of osteogenic and adipogenic differentiation potential of MSCs, fibroblasts, and chondrocytes. Supplementary Figure 8: cell viability of BM-MSCs cultivated under serum-free conditions in agarose hydrogel. Supplementary Figure 9: validation of anti-pNIIB52 for flow cytometry analysis of type IIB procollagen expression.
