# Supplementary material for: Characterization of Different Sources of Human MSCs Expanded in Serum-Free Conditions with Quantification of Chondrogenic Induction in 3D
Source: Stem Cells Int. 2019 Jun 20;2019:2186728. doi: 10.1155/2019/2186728 (PMC6610765; doi:10.1155/2019/2186728)

Supplementary Materials

Supplementary Figure 1: List of antibodies used for the immunophenotypic analysis.

Supplementary Figure 2: Presentation of the different combinations of antibodies coupled to fluorochromes that were used for the immunophenotypic analysis. The fluorochromes and their excitation wavelength are indicated in color. In a first screening panel, 27 markers were examined by dispatching combinations of antibodies over 6 tubes (top table). In a second screening panel, 4 additional markers were analyzed in a single tube (bottom table).

Supplementary Figure 3: Representative flow cytometric histograms of "classical", exclusion and "advanced characterization" markers expressed on BM-MSCs cultivated at passage 1, under serum free conditions. Percentage of positive cells is represented as mean ± SD. (*blue*: isotype control, *black*: FMO control, *red*: sample).

Supplementary Figure 4: Oligonucleotide primers used for the real-time polymerase chain reaction analyses. The references of the studies where the primers have been designed are indicated.

Supplementary Figure 5: Growth characteristics of MSCs and fibroblasts during long-term expansion under serum-free conditions. The doubling times are compared from passage 1 to passage 10 (n = 5 for BM-MSCs, n = 6 for WJ-MSCs, n = 8 for DP-MSCs, n = 4 for AT-MSCs and n = 3 for fibroblasts). Error bars: mean ± SD.

Supplementary Figure 6: Immunophenotype of MSCs, fibroblasts and articular chondrocytes cultivated under serum-free conditions. The expression of 31 markers was analyzed by flow cytometry in cell preparations from passage 1. The values represent the percentage of positive cells and are reported as mean with standard deviation (SD). N represents the number of donors. These values correspond to the values reported in the histograms presented in Figure 2.

Supplementary Figure 7: Evaluation of osteogenic and adipogenic differentiation potential of MSCs, fibroblasts and articular chondrocytes. Cells were amplified for 2 passages in serum-free culture medium then induced for 21 days for osteogenic or adipogenic differentiation, in presence of 10% FBS. (a) Upper panel: after culture in osteoblastic induction medium, calcium mineralization was demonstrated by alizarin red S staining. A representative example from three experiments is shown (n = 3). Lower panel: quantification of alizarin red S staining and relative mRNA expression of *RUNX2* and *ALPL* is shown, relative to non-induced, control cells (reference value = 1). (b) Upper panel: after culture in adipogenic induction medium, lipid droplets are revealed in the cytoplasm with oil red O staining. Note the absence of lipid droplets in the fibroblast population. A representative example from three experiments is shown (n = 3). Lower panel: quantification of oil red O staining and relative mRNA expression of *LEP* and *PPARG* is shown, relative to non-induced, control cells (reference value = 1). Error bars: mean ± SD (n = 3). * Indicates statistically significant differences between induced cells and control cells (*p < 0.05, **p < 0.01 and ***p < 0.001). The scale bar corresponds to 100 µm.

Supplementary Figure 8: Cell viability of BM-MSCs cultivated under serum-free conditions in agarose hydrogel. P1 BM-MSCs amplified in serum-free SPE-IV medium were embedded in agarose hydrogel. The constructs were then cultivated in serum-free chondrogenic BTβ3 medium (containing high glucose DMEM, 1 mM sodium pyruvate, 50 µg/mL L-ascorbate-2-phosphate, 0.1 µM dexamethasone, 1% ITS, 50 ng/mL BMP-2, 10 ng/mL TGF-β3) alone (a) or supplemented with 5% serum-free SPE-IV medium (b). The cell viability was evaluated by using the Cellstain double staining kit (dead cells stain red and live cells stain green). Supplementation of the chondrogenic medium with 5% SPE-IV medium maintained good viability of BM-MSCs. The photographs shown here correspond to a 10-day culture period and are representative of three experiments. The scale bar corresponds to 80 µm.

Supplementary Figure 9: Validation of anti-pNIIB52 for flow cytometry analysis of type IIB procollagen expression. Nasal chondrocytes were used in these experiments. (a) Chondrocytes were amplified then cultivated for 21 days in monolayer in the presence of the chondrogenic BTβ3 cocktail, to allow chondrocyte redifferentiation. The chondrocytes were fixed, permeabilized and a titration assay was performed by incubating chondrocytes with different concentrations of anti-pNIIB52 labeled with Alexa Fluor 647 dye, in order to detect IIB expression with optimal signal to noise ratio. A representative titration assay is shown and 0.35 µg/1 x 10^6 cells was the titer selected for all subsequent flow cytometry analyses. Red dots: stained samples, blue dots: unstained samples. (b) Analysis of type IIB expression by using fluorescently labeled anti-pNIIB52 in chondrocytes cultivated in different culture conditions. After amplification in monolayer, chondrocytes were negative to IIB procollagen whereas redifferentiation triggered by 21 days of BTβ3 treatment resulted in 37.6 ± 4.2% or 94.6 ± 2.1% of positive cells, for chondrocytes redifferentiated in monolayer or in agarose, as

indicated. (c) Demonstration of staining specificity of anti-pNIIB52 in flow cytometry analysis. The immunizing peptide that was shown to block anti-pNIIB52 in our original Western-blotting and immunohistochemistry analyses was used here similarly in peptide competition assay. Chondrocytes amplified and redifferentiated in agarose as described in (B) were stained with anti-pNIIB52 or with anti-pNIIB52 pre-incubated with an excess of the immunizing peptide. No expression of type IIB procollagen was detected when anti-pNIIB52 was first blocked with the immunizing peptide. Percentage of positive cells is represented as mean ± SD (n = 3).

Supplementary Figure 1

Target	Format	Vendor	Isotype	Reference
CD10	PE	BD Biosciences	IgG2a, κ	555375
CD13	APC	BD Biosciences	IgG1, κ	557454
CD14	APC-H7	BD Biosciences	IgG2b, κ	560180
CD15	V450	BD Biosciences	IgM, κ	642917
CD29	APC	BD Biosciences	IgG1, κ	559883
CD31	FITC	BD Biosciences	IgG1, κ	555445
CD31	APC-Cy7	Biolegend	IgG1, κ	303120
CD33	V450	BD Biosciences	IgG2a, κ	561157
CD34	APC	BD Biosciences	IgG1, κ	555824
CD34	PE-CF594	BD Biosciences	IgG1, κ	562383
CD44	APC-H7	BD Biosciences	IgG2b, κ	560532
CD45	V500	BD Biosciences	IgG1, κ	560777
CD45	BV510	BD Biosciences	IgG1, κ	563204
CD49a	PE	BD Biosciences	IgG1, κ	559596
CD56	V450	BD Biosciences	IgG1, κ	560360
CD56	AF700	Biolegend	IgG1, κ	318316
CD63	FITC	BD Biosciences	IgG1, κ	557288
CD73	PE-Cy7	BD Biosciences	IgG1, κ	561258
CD79a	BV421	BD Biosciences	IgG1, κ	562852
CD90	FITC	BD Biosciences	IgG1, κ	555595
CD105	PE	BD Biosciences	IgG1, κ	555487
CD106	FITC	BD Biosciences	IgG1, κ	551146
CD133	PE	Miltenyi biotec	IgG2b	130-090-853
CD146	FITC	BD Biosciences	IgG1, κ	560846
CD146	AF488	Biolegend	IgG2a, κ	342008
CD166	PE	BD Biosciences	IgG1, κ	559263
CD184	APC	BD Biosciences	IgG2a, κ	555976
CD271	PerCP-Cy5.5	BD Biosciences	IgG1, κ	560834
CD271	PE-Cy7	BD-Biosciences	IgG1, κ	562852
CD340	PerCP-Cy5.5	Biolegend	IgG1, κ	324416
D7-Fib	PE	Antibodies-online	IgG2a, κ	ABIN319868
HLA ABC	V450	BD Biosciences	IgG1, κ	561346
HLA DR	V500	BD Biosciences	IgG1, κ	561224
HLA G	APC	eBioscience	IgG2a, κ	17-9957-42
MSCA-1	APC	Biolegend	IgG1, κ	327308
Stro-1	AF647	Biolegend	IgM, λ	340104
Stro-1	PE	Santa Cruz	IgM, λ	sc-47733 PE
α10 integrin	AF488	Xintela Ab.	IgG2a	NA

Supplementary Figure 2

Laser	PMT	Channel	tubes											
			1		2		3		4		5		6	
Blue 488 nm	E	1	CD90	FITC	CD106	FITC	CD63	FITC	CD146	FITC	CD31	FITC	CD90	FITC
	D	2	CD133	PE	CD105	PE	CD166	PE	CD10	PE	D7-Fib	PE	CD49a	PE
	B	3	CD271	PerCP-Cy5.5	CD271	PerCP-Cy5.5	CD271	PerCP-Cy5.5	CD271	PerCP-Cy5.5	7-AAD	7-AAD	CD271	PerCP-Cy5.5
	A	4	CD73	PE-Cy7	CD73	PE-Cy7	CD73	PE-Cy7	CD73	PE-Cy7	CD73	PE-Cy7	CD73	PE-Cy7
Red 633 nm	C	5	CD34	APC	HLA-G	APC	Stro-1	AF647	CD13	APC	CD29	APC	CD184	APC
	A	6	CD44	APC-H7	CD14	APC-H7	CD44	APC-H7	CD44	APC-H7	CD44	APC-H7	CD44	APC-H7
Violet 405 nm	B	7	CD33	V450	HLA-ABC	V450	CD56	V450	CD56	V450	CD15	V450	CD15	V450
	A	8	CD45	V500	HLA-DR	V500	CD45	V500	CD45	V500	CD45	V500	CD45	V500

Laser	PMT	Channel	tube	
			7	
Blue 488 nm	E	1	α10 ITG	AF488
	D	2	Stro-1	PE
	B	3	CD340	PerCP-Cy5.5
	C	4	CD34	PE-CF594
	A	5	CD271	PE-Cy7
Red 633 nm	C	6	MSCA-1	APC
	B	7	CD56	AF700
	A	8	CD31	APC-Cy7
Violet 405 nm	B	9	CD79a	BV421
	A	10	CD45	BV510

Supplementary Figure 3

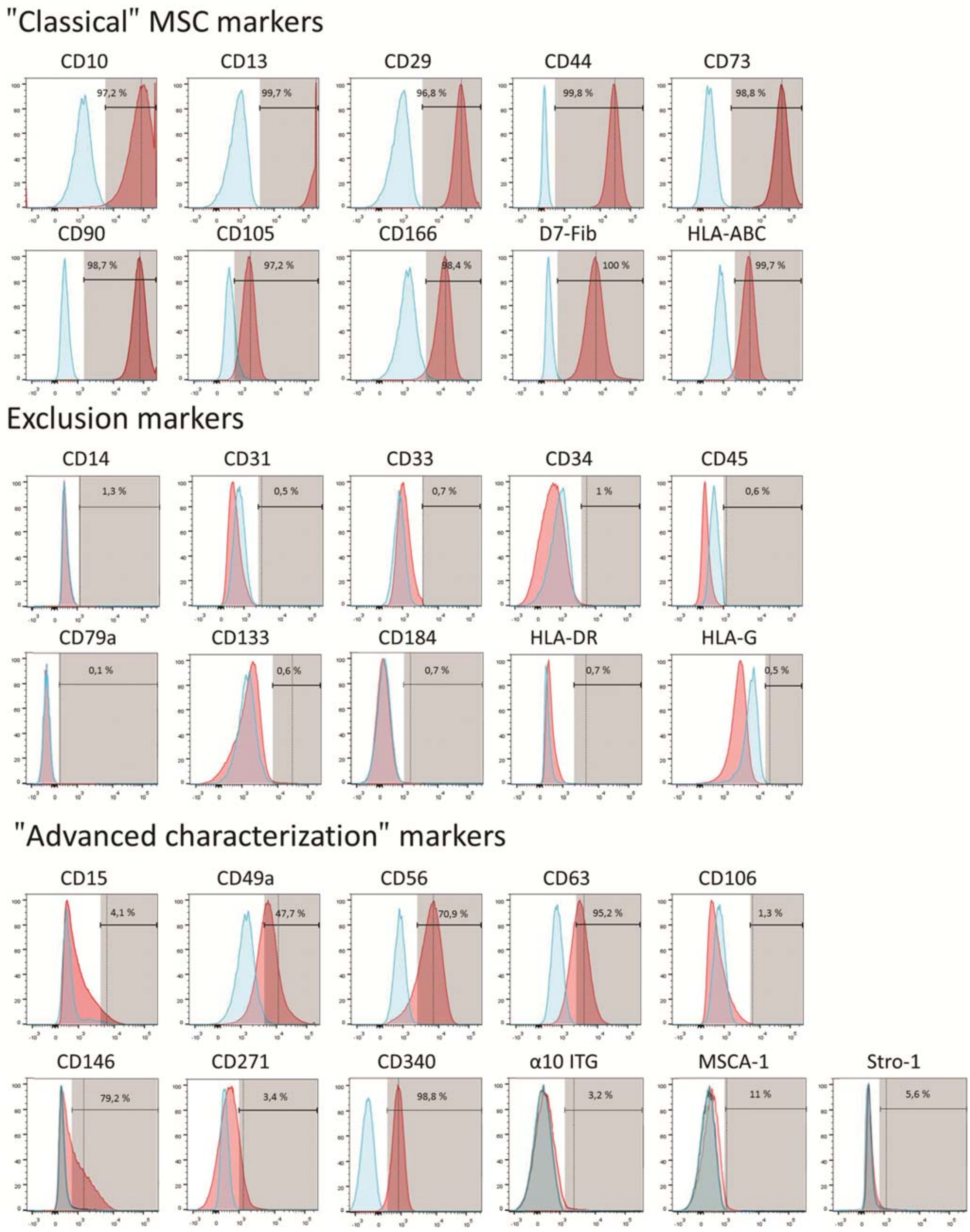

Supplementary Figure 4

Genes	Primer sequence (5'-3') (F : Forward ; R : Reverse)	References
COL2A1	*F : GGCAATAGCAGGTTCACGTACA*<br>*R : CGATAACAGTCTTGCCCCACT*	Martin *et al.* [76]
ACAN	*F : TCGAGGACAGCGAGGCC*<br>*R : TCGAGGGTGTAGCGTGTAGAGA*	Martin *et al.* [76]
COL1A1	*F : CAGCCGCTTCACCTACAGC*<br>*R : TTTTGTATTCAATCACTGTCTTGCC*	Martin *et al.* [76]
COL10A1	*F : CAAGGCACCATCTCCAGGAA*<br>*R : AAAGGGTATTTGTGGCAGCATATT*	Martin *et al.* [76]
MMP13	*F : TCCTCTTCTTGAGCTGGACTCATT*<br>*R : CGCTCTGCAAACTGGAGGTC*	Ronzière *et al.* [52]
ALPL	*F : GACCTCGTTGACACCTGGAAG*<br>*R : TTCCTGTTCAGCTCGTACTGC*	Rodriguez *et al.* [55]
RUNX2	*F : ACCAGATGGGACTGTGGTTAC*<br>*R : AGACGGTTATGGTCAAGGTG*	Rodriguez *et al.* [55]
LEP	*F : CACCAGGATCAATGACATTTC*<br>*R : TGCCAGTGTCTGGTCCATCTTG*	Rodriguez *et al.* [55]
PPARG	*F : TCTCTCCGTAATGGAAGACC*<br>*R : GCATTATGAGACATCCCCAC*	Rodriguez *et al.* [55]
GUSB	*F : TGGTTGGAGAGCTCATTTGG*<br>*R : CTCTCGCAAAAGGAACGCTG*	Rodriguez *et al.* [55]
GAPDH	*F : ATGGGGAAGGTGAAGGTCG*<br>*R : TAAAAGCAGCCCTGGTGACC*	Martin *et al.* [76]

Supplementary Figure 5

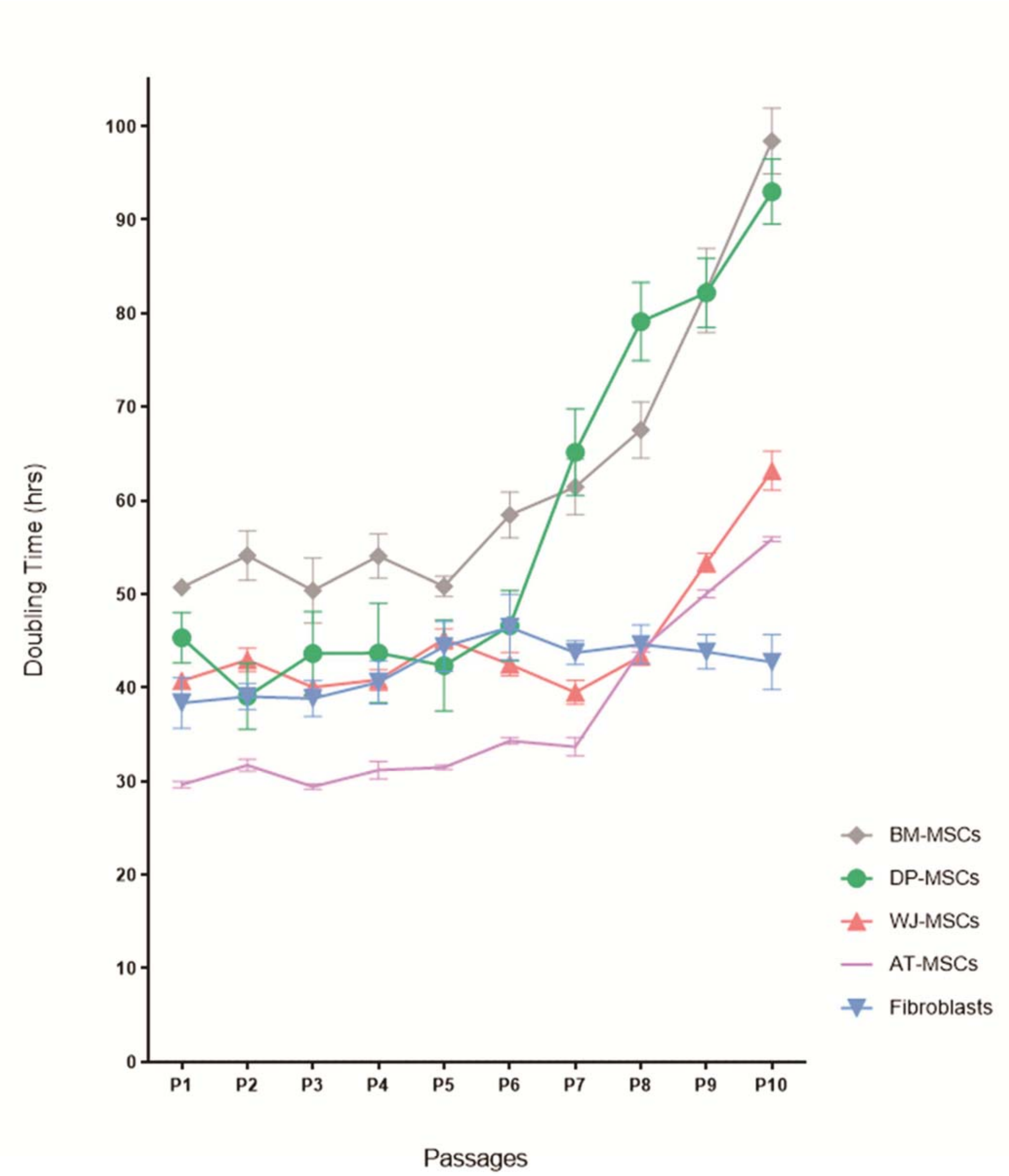

Supplementary Figure 6

		BM-MSCs			WJ-MSCs			DP-MSCs			AT-MSCs			Fibroblasts			Chondrocytes		
		Mean	SD	N	Mean	SD	N	Mean	SD	N	Mean	SD	N	Mean	SD	N	Mean	SD	N
"Classical" MSC markers	CD10	**97.2**	5.7	8	**98.9**	2.4	5	**96.5**	5,0	5	**100,0**	0.1	5	**93.2**	1.7	3	**4.6**	6.4	3
	CD13	**99.7**	0.6	8	**96.3**	5.3	5	**100,0**	0,0	5	**100,0**	0.1	5	**11.7**	12.4	3	**72,0**	0.8	3
	CD29	**96.8**	4,0	8	**99.6**	1.1	5	**99,0**	0.9	5	**100,0**	0,0	5	**99.6**	0.3	3	**100,0**	0.1	3
	CD44	**99.8**	0.9	8	**99.8**	0.7	5	**100,0**	0,0	5	**100,0**	0.1	5	**100,0**	0,0	3	**90.5**	10,0	3
	CD73	**98.8**	3.2	8	**99.7**	0.5	5	**99.1**	1.2	5	**99.8**	0.4	5	**95.9**	6.8	3	**92.3**	11,0	3
	CD90	**98.7**	3.4	8	**99.8**	0.5	5	**98.3**	2.2	5	**99.7**	0.5	5	**95.6**	6.8	3	**99.4**	0.6	3
	CD105	**97.2**	6.3	8	**99.9**	0.2	5	**100,0**	0,0	5	**98,0**	3.5	5	**100,0**	0,0	3	**56.2**	13.7	3
	CD166	**98.4**	3.4	8	**99.2**	1.3	5	**100,0**	0,0	5	**95.5**	7.3	5	**100,0**	0.1	3	**51.2**	23.3	3
	D7-Fib	**100,0**	0,0	8	**99.8**	0.3	5	**99.9**	0.1	5	**99.7**	0.5	5	**100,0**	0.1	3	**48.3**	15.4	3
	HLA-ABC	**99.7**	0.5	8	**99.9**	0.1	5	**100,0**	0,0	5	**98.4**	3.2	5	**100,0**	0,0	3	**61.9**	14.1	3
Exclusion markers	CD14	**1.3**	1.1	8	**1.1**	0.6	5	**0.5**	0.2	5	**0.8**	0.7	5	**1,0**	0.2	3	**1.1**	0.5	3
	CD31	**0.5**	0.4	8	**0.7**	0.8	5	**0.4**	0.2	5	**0.5**	0.6	5	**0.1**	0,0	3	**0.3**	0.2	3
	CD33	**0.7**	0.5	8	**0.6**	0.8	5	**0.3**	0.3	5	**0.4**	0.4	5	**0.1**	0.1	3	**1.5**	0.2	3
	CD34	**1,0**	0.9	8	**0.7**	0.5	5	**0.5**	0.2	5	**0.5**	0.5	5	**0.1**	0.1	3	**0.1**	0.1	3
	CD45	**0.6**	1.1	8	**0.8**	2.1	5	**0.8**	0.4	5	**0.5**	0.7	5	**0.1**	0.1	3	**0.6**	0.3	3
	CD79a	**0.1**	0.5	8	**0.2**	0.6	5	**0.5**	0.5	5	**0.2**	0.1	5	**0.4**	0.1	3	**0.2**	0.4	3
	CD133	**0.6**	0.3	8	**0.6**	0.7	5	**0.7**	0.1	5	**0.4**	0.2	5	**0.1**	0,0	3	**0.5**	0.4	3
	CD184	**0.7**	1.2	8	**0.8**	0.6	5	**0.7**	0.3	5	**0.2**	0.2	5	**0.2**	0.1	3	**0.1**	0.1	3
	HLA-DR	**0.7**	0.4	8	**0.8**	0.5	5	**0.5**	0.6	5	**0.9**	0.8	5	**0.1**	0.1	3	**0.4**	0.6	3
	HLA-G	**0.5**	0.4	8	**0.8**	0.4	5	**0.9**	0.1	5	**0.4**	0.6	5	**0.1**	0.1	3	**0.9**	0.5	3
"Advanced characterization" markers	CD15	**4.1**	2.3	8	**4,0**	5.3	5	**5.5**	3.9	5	**16.4**	10.1	5	**93.2**	3.3	3	**2.2**	1.1	3
	CD49a	**47.7**	12.2	8	**65.5**	16.1	5	**99.4**	1,0	5	**54.9**	26.2	5	**99.9**	0.1	3	**54.6**	18.7	3
	CD56	**70.9**	29.3	8	**61.2**	13.3	5	**24.2**	3.4	5	**4.6**	4.4	5	**1,0**	0.8	3	**0.5**	0.4	3
	CD63	**95.2**	10.2	8	**86.9**	13.9	5	**99.8**	0.1	5	**86.2**	9.7	5	**100,0**	0.1	3	**77.4**	3.8	3
	CD106	**1.3**	1.3	8	**1,0**	1,0	5	**1.3**	1.2	5	**0.7**	0.9	5	**25.7**	25.1	3	**0.9**	1,0	3
	CD146	**79.2**	15.4	8	**36.9**	23.7	5	**41.7**	17,0	5	**6.6**	5.8	5	**1.3**	0.6	3	**0.9**	1.2	3
	CD271	**3.4**	1.9	8	**3.2**	1.2	5	**0.6**	0.4	5	**1.9**	2.5	5	**0.1**	0.1	3	**0.8**	0.6	3
	CD340	**98.8**	3.2	8	**98.8**	3.2	5	**96.1**	2.9	5	**25.8**	11.4	5	**0.6**	0.4	3	**51.2**	11,0	3
	α10 ITG	**3.2**	1.3	3	**1.2**	0.4	5	**1.4**	0.6	5	**0.6**	0.1	3	**0,6**	0.2	3	**0.7**	0.2	3
	MSCA-1	**11,0**	1.8	5	**5.8**	1.1	5	**17.0**	2.9	5	**4,0**	3.9	3	**5.3**	0.5	3	**7.7**	3.6	3
	Stro-1	**5.6**	1.4	8	**1.3**	0.9	5	**0.3**	0.1	5	**0.4**	0.3	5	**0.8**	0.6	3	**1.1**	1.6	3

Supplementary Figure 7

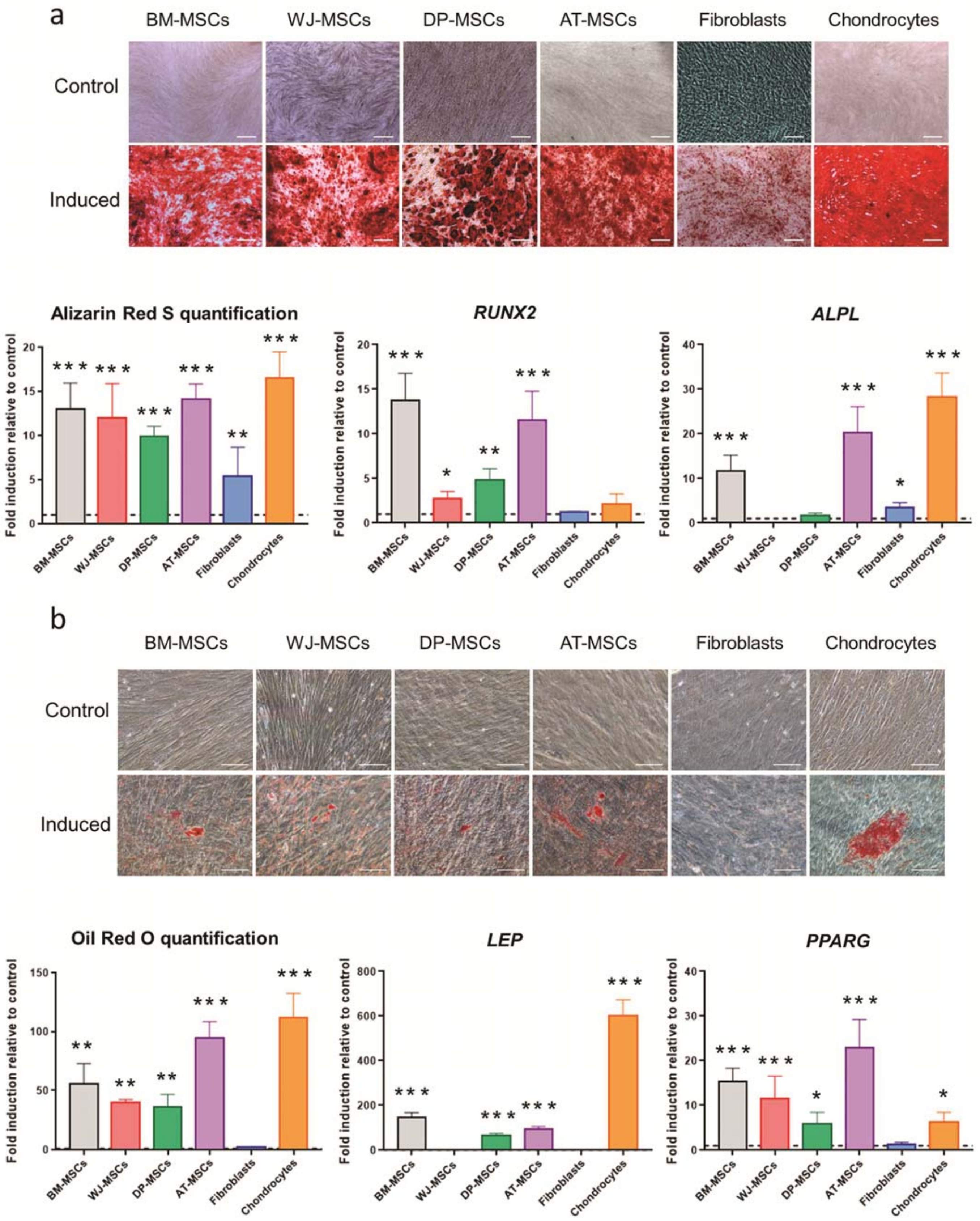

Supplementary Figure 8

a
DMEM High Glucose
+ 1 % ITS + BTβ3

b
DMEM High Glucose
+ 1 % ITS + 5% SFM + BTβ3

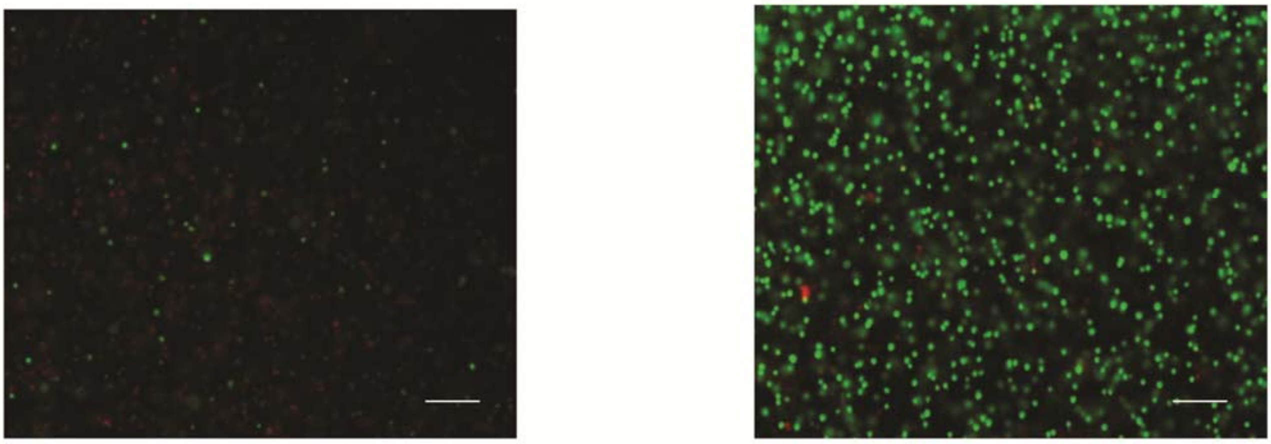

Supplementary Figure 9

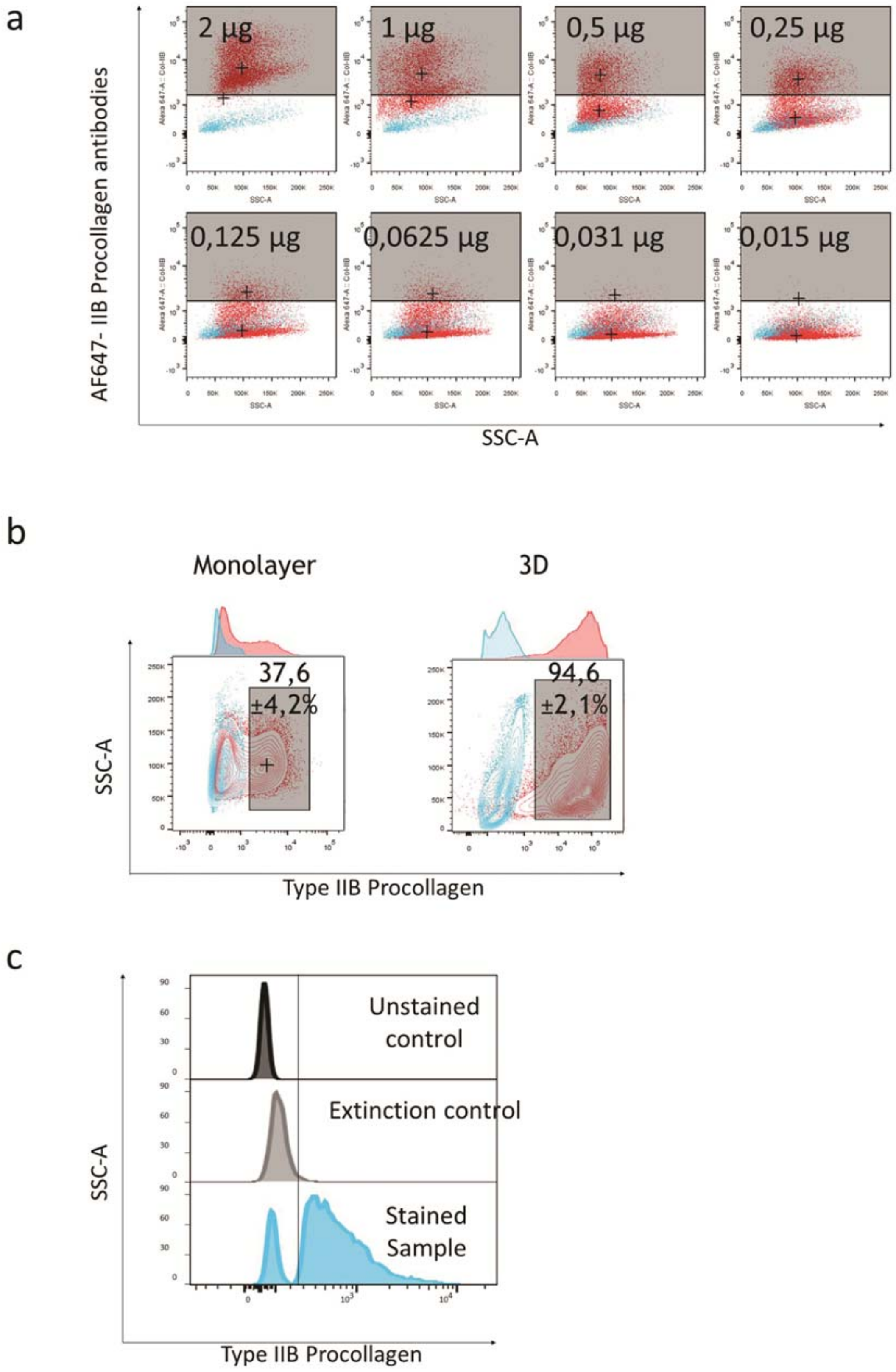

Supplement: Supplementary Materials — Supplementary Figure 1: list of antibodies used for the immunophenotypic analysis. Supplementary Figure 2: presentation of the different combinations of antibodies coupled to fluorochromes that were used for the immunophenotypic analysis. Supplementary Figure 3: representative flow cytometric histograms of “classical,” exclusion, and “advanced characterization” markers expressed on BM-MSCs cultivated at passage 1, under serum-free conditions. Supplementary Figure 4: oligonucleotide primers used for the real-time polymerase chain reaction analysis [76]. Supplementary Figure 5: growth characteristics of MSCs and fibroblasts during long-term expansion under serum-free conditions. Supplementary Figure 6: immunophenotype of MSCs, fibroblasts, and chondrocytes cultivated under serum-free conditions. The expression of 31 markers was analyzed by flow cytometry. Supplementary Figure 7: evaluation of osteogenic and adipogenic differentiation potential of MSCs, fibroblasts, and chondrocytes. Supplementary Figure 8: cell viability of BM-MSCs cultivated under serum-free conditions in agarose hydrogel. Supplementary Figure 9: validation of anti-pNIIB52 for flow cytometry analysis of type IIB procollagen expression. [file 2186728.f1.pdf]
